# Pipe vibration attenuation through internal damping and optimal design of vibro-impact systems

**DOI:** 10.1038/s41598-023-33640-y

**Published:** 2023-04-20

**Authors:** Fabrizio Aloschi, Roberto Andreotti, Oreste Salvatore Bursi

**Affiliations:** 1 Department of Civil, Environmental and Mechanical Engineering, University of Trento, Via Mesiano 77, 38123 Trento, Italy; 2 IGF - Ingenieurgesellschaft Dr. Ing. Fischbach mbH, An Der Vogelrute 2, 50374 Erftstadt-Lechenich, Germany

**Keywords:** Civil engineering, Mechanical engineering

## Abstract

Pipelines periodically supported by rack structures (PPRs) are common in chemical and petrochemical plants, among others, and conventional tools such as dampers and hysteretic absorbers are commonly used to mitigate large vibrations in these systems. In this study, we explore two alternative strategies: (i) enhancing the attenuation rate of PPR vibrations through structural internal damping, and (ii) using nonlinear vibro-impact systems (VIS) to reduce seismic vibrations in a PPR. To shed light on the first strategy, we develop analytical dispersion relations for a PPR and show how damping can improve the mitigation capabilities of the periodic system. As for the second strategy, we consider a 9-node beam, i.e., a single span (SS) of a PPR equipped with a VIS, and combine the central composite design (CCD) and Kriging metamodelling to maximize dissipation energy and minimize the number of impacts. This multi-objective optimization problem aims to find the most effective design solution for the VIS in terms of gap and coefficient of restitution (COR). Additionally, we consider the stochastic nature of seismic input and the possible chaotic behavior of the VIS. To account for the sensitive variability of the number of impacts in seismic records, we perform incremental dynamic analyses and calculate fragility functions for various engineering demand parameters, including the number of impacts. We define a 3D surface for selecting the optimal gap-COR pair. When impacts occur, transient results can be chaotic, and we compute the largest Lyapunov exponents of a few representative trajectories.

## Introduction

Pipelines supported by rack structures (PPRs) are a crucial means of carrying liquefied gas in different types of plants, such as liquefied natural gas plants, thermal power plants, petroleum industries, and chemical plants. These structures have demonstrated vulnerability to excessive vibrations^1–3^, such as ground-borne vibrations^4–9^ and flow-induced turbulence^10–13^. To mitigate vibrations, researchers have exploited bandgaps and attenuation zones exerted by periodically arranged structures. In some research works, Bloch waves have been applied to long, distributed infrastructures, such as bridges^14,15^, and pipes^16–18^. The dispersion analysis of Iqbal et al.^16^ revealed the dynamic flexural behavior of long supported pipes modeled as an infinite periodic structure. However, none of these works took into account the supplemental dissipation that damping^19,20^ may provide to continuous systems.

Researchers have also used linear and nonlinear external tools to attenuate large vibrations, such as pounding tuned mass dampers (PTMD)^21^, pipe-in-pipe systems^22^, multi-stage dampers^23^, and Stockbridge dampers^24^. Song et al.^21^ developed a numerical analysis based on the Hertzian contact to model the pounding force and performed experimental tests on a pipeline coupled with a PTMD. The presence of the PTMD increased the damping ratio and effectively induced amplitude attenuation; nonetheless, this device is complicated to design since it combines vibro-impact and TMD. In this work, we exploit the impact phenomenon^26–41^ with a nonlinear device that constrains the amplitude displacement of the pipe's interface.

Conventional linear dampers, such as TMDs, are not always recommended for vibration mitigation due to their low performance under certain conditions caused by detuning effects^25^. Moreover, they may not be sufficient to mitigate the abrupt flow-induced increases of velocity. In such cases, impact-based dissipation systems serve as a powerful alternative^26^. However, high-velocity impacts can cause large deformations near the contact area, eventually leading to system design difficulties. Therefore, the design optimization of dissipative vibro-impact systems (VIS) needs further study. To the best of our knowledge, it is difficult to estimate the frequency response function of a nonlinear VIS and compare it with that of a TMD system, as a VIS often exhibits non-periodic motion. Additionally, TMDs have limitations in terms of their ability to suppress a broader range of frequencies^27,28^, and as such, nonlinear systems have become the preferred choice for this purpose. Hysteretic quadratic nonlinearity devices^29^, energy sinks^30,31^, nonlinear vibration absorbers^32,33^, and other nonlinear systems have proven to be effective in suppressing broader frequency ranges.

**Figure 1:**
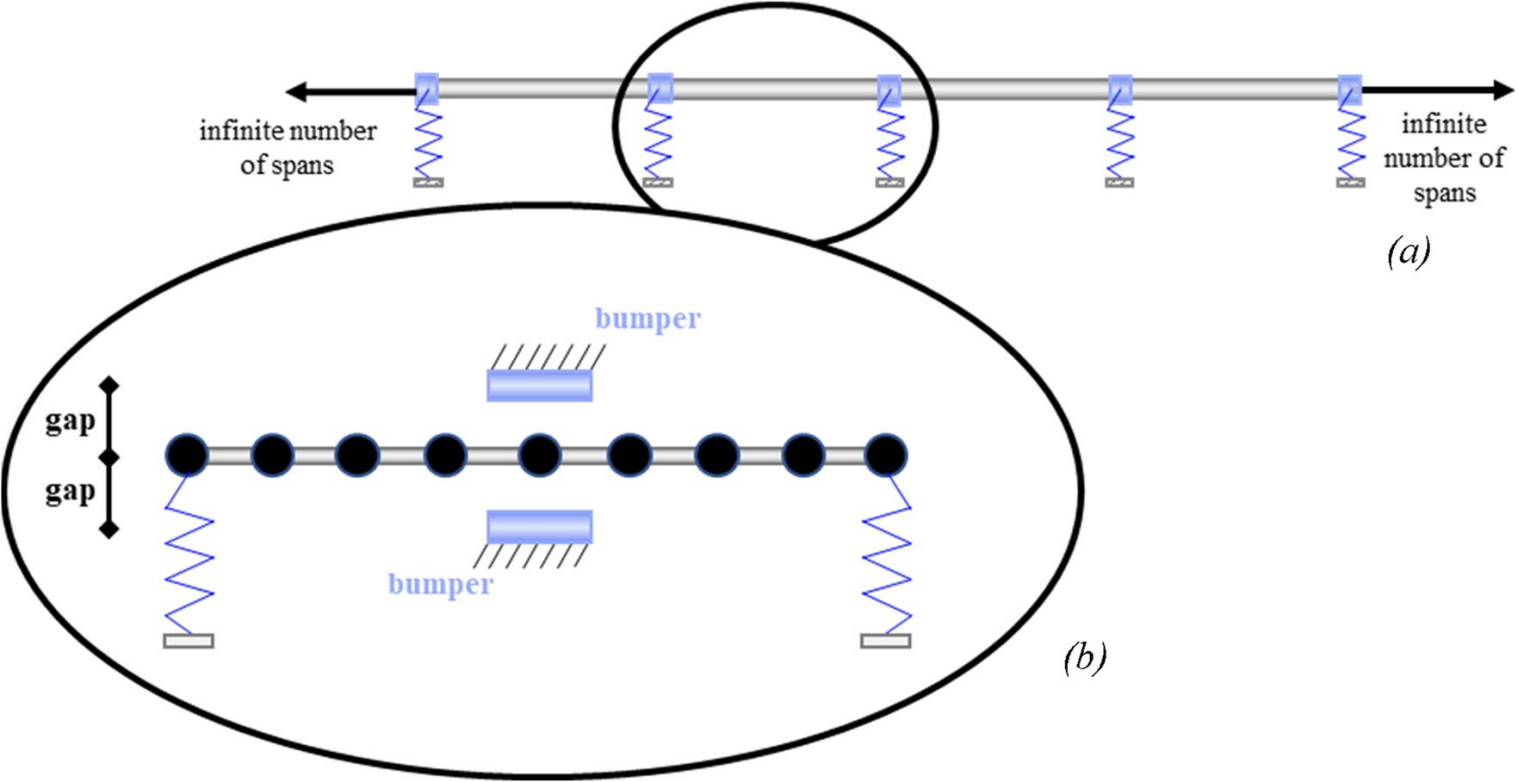
(**a**) Periodic damped pipeline on flexible supports and (**b**) single span (SS) endowed with a vibro-impact system (VIS) for impact-based energy dissipation.

Within this framework, the mitigation of dynamic vibration in PPRs is performed by means of two main strategies: (i) the attenuation properties of PPR models by means of internal damping; (ii) the optimized performance of a VIS subjected to stochastic (seismic) loading. Figure 1 schematically depicts the two strategies employed.

In particular, Fig. 1a shows a linear PPR; the attenuation zones due to internal damping will be obtained by applying dispersion analysis to a single cell. Figure 1b depicts, instead, a single span (SS) of a pipeline equipped with the VIS. The relevant nonlinear dissipation system is optimized in terms of maximum dissipation energy and minimum number of impacts when subjected to stochastic excitations. To model the impact phenomenon, a non-smooth approach is adopted with an algorithm that combines instantaneous and finite duration contact^34–36^; pipes and bumpers are assumed to be rigid bodies, and the COR is given by Newton’s impact law. To further streamline the computational effort, we adopt the CCD, along with the Kriging metamodel implemented in UQLab^43^. Both Kriging metamodelling and response surface methodologies (RSM) in general^44^ provide the designer with an overall perspective of the system's response within the design domain, which in this paper are the gap and the COR. RSM is indeed a common tool for design optimization problems^45–49^, especially for those cases where the input is (seismic) stochastic, and the system’s response is nonlinear. In this respect, among many interesting features of the dynamics of impacts, bifurcation and chaos have gained much attention over the last few decades. Two basic characteristics of chaos as a dynamical state are commonly discussed^39–42^: (i) the chaos pertains to a pseudorandom behavior observed in a deterministic nonlinear dynamical system, i.e., the system's output appears to be statistically random despite having been produced by a deterministic and repeatable process; (ii) the chaos causes a sensitive dependence on initial conditions, thus the initial states of close trajectories exponentially separate; the rate of separation over time is identified by the Lyapunov exponent. Shaw^39^ experimentally tested an elastic beam with a one-sided displacement constraint under periodic excitation and reported nonlinear features of the beam, such as subharmonic resonances, period doublings, and chaotic regimes. The results were validated with the analytical model of a single oscillator with periodic excitation and a piecewise linear restoring force. Coexistent attractors and multi-stability appeared in the impact oscillator of Costa et al.^41^, and the existence of chaos was proven with the 0–1 test and the Lyapunov exponents^42^.

Nonetheless, the design problem of a chaotic deterministic system under (seismic) stochastic loading, such as earthquakes, still needs to be deepened. To handle this task, the design optimization procedure of a pipe with vibro-impact is complemented with a fragility assessment. Fragility functions usually derive from a variety of approaches like static structural analyses, judgment, or field observations of damage. In this work, we derive the so-called analytical fragility functions from incremental dynamic analyses (IDA)^50–52^. Given the unpredictability of the number of impacts due to chaos, the fragility function will be defined by a surface^53^ for a variety of damage states (DM). Just to prove the existence of chaos in the SS depicted in Fig. 1b, a few chaotic trajectories and the bifurcation diagrams are reported in the *Supplementary Information*. They have been evaluated for certain ranges of frequencies in the vicinity of the natural frequency of the SS, and the largest Lyapunov exponents have been estimated. Due to the nonlinear dependence of the dispersion features upon amplitude and frequency of excitation, this last task has required periodic excitation with multiple amplitudes.

## Methods

### Internal and external damping models in periodic* PPRs*

**Figure 2:**
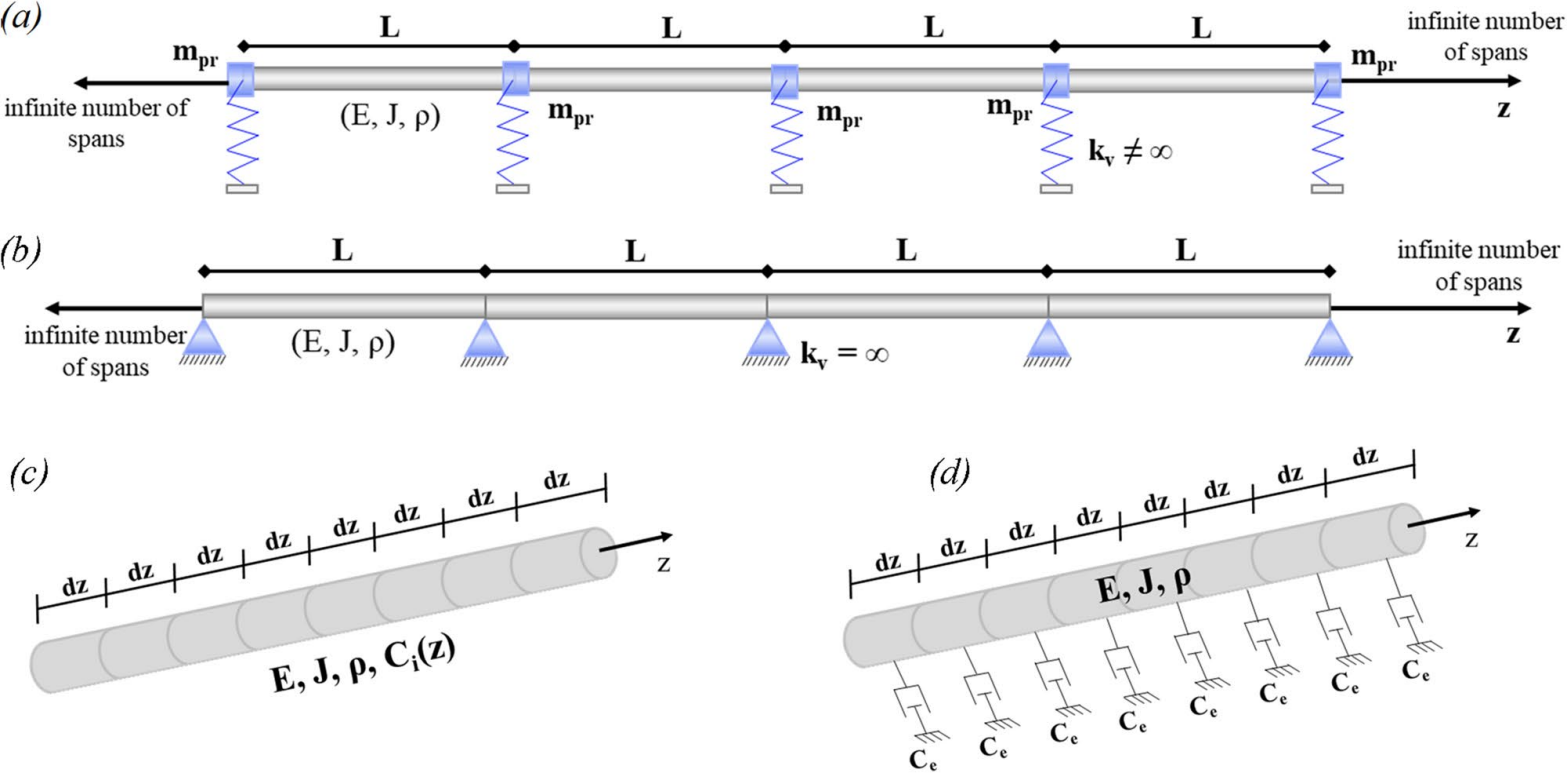
(**a**) PPR_f_: periodic damped pipeline coupled with the flexible supports and the masses m_pr_ of a pipe rack; (**b**) PPR_r_: periodic damped pipeline on rigid supports; (**c**) internal or structural damping model for a continuous beam and (**d**) external viscous damping model.

To improve the attenuation properties of the PPRs depicted in Fig. 2 both damping and periodic properties are employed.

The supports of the pipelines depend on the flexural stiffness *k*_*v*_ of the pipe rack’s columns. In Fig. 2a, flexible springs support the periodic pipeline. Figure 2b depicts the model of a pipeline supported by rigid supports. Hereinafter, the models in Fig. 2a, b are called PPR_f_ and PPR_r_, respectively. The damping models proposed to enhance vibration mitigation are linear and have different sources: internal material damping due to localized plastic deformation within the apparent elastic limit, see Fig. 2c; and the external viscous damping that is proportional to the forcing frequency, see Fig. 2d. Since the length ${\text{dz }} \to { 0}$, the damping is continuously distributed along the pipe. Therefore, internal and external damping forces can be represented by two different equations for the Euler–Bernoulli beam.

For the PPR_f_ and the internal damping model the Euler–Bernoulli equation reads,

1 $$ \frac{{\partial^{{2}} }}{{\partial {\text{z}}^{{2}} }}\left[ {{\text{EJ}}\frac{{\partial^{{2}} {\text{w(z,t)}}}}{{\partial {\text{z}}^{{2}} }}} \right]{ + }\frac{{\partial^{{2}} }}{{\partial {\text{z}}^{{2}} }}\left[ {{\text{C}}_{{\text{i}}} \frac{{\partial^{{2}} \frac{{\partial {\text{ w(z,t)}}}}{{\partial {\text{t}}}}}}{{\partial {\text{z}}^{{2}} }}} \right]{ + }\rho {\text{A}}\frac{{\partial^{{2}} {\text{w(z,t)}}}}{{\partial {\text{t}}^{{2}} }}{ = 0, } $$

where *ρ, E, A* and *J* are, respectively, the material density, the Young modulus, the area and the inertia of the beam cross section. The term *w*(*z,t*) is the beam transversal displacement. *C*_*i*_ is the internal damping coefficient. Instead, for the externally damped PPR_f_, the Euler–Bernoulli equation reads,

2 $$ \frac{{\partial^{{2}} }}{{\partial {\text{z}}^{{2}} }}\left[ {{\text{EJ}}\frac{{\partial^{{2}} {\text{w(z,t)}}}}{{\partial {\text{z}}^{{2}} }}} \right]{\text{ + C}}_{{\text{e}}} \frac{{\partial {\text{ w(z,t)}}}}{{\partial {\text{t}}}}{ + }\rho {\text{A}}\frac{{\partial^{{2}} {\text{w(z,t)}}}}{{\partial {\text{t}}^{{2}} }}{ = 0,} $$

where C_e_ is the external damping coefficient. In (1) and (2) the damping coefficients read, respectively,

3 $$ {\text{C}}_{{\text{i}}} { = }\frac{{{2}\zeta_{{\text{i,n}}} {\text{ EJ}}}}{{\omega_{{\text{n}}} }}{,} $$

4 $$ {\text{C}}_{{\text{e}}} { = 2}\zeta_{{\text{e,n}}} { }\rho {\text{A}}\;\omega_{{\text{n}}} {,} $$

where *ζ*_*i/e*_ is the modal damping ratio; *i/e* specifies internal or external damping and *ω*_*n*_ defines the modal frequency. Note that the two coefficients* C*_*i/e*_ have different physical meanings and are dimensionally different. *C*_*i*_ in (3) comes from the assumption that structural internal damping does not entail plastic deformations in the cross section of the pipe^19^. In this regard, Kimball et al.^20^ showed that, for metals subjected to cyclic stress, internal friction entails strains that remain below the elastic limit. *C*_*e*_ in (4), instead, represents the common proportionality constant of a viscous damping model.

The mass and the stiffness of the pipe are constant along its length. Since the systems are periodic, we consider (1) and (2) and apply the Floquet–Bloch theorem to the *j*th support,

5 $$ \psi_{{\text{i/e}}} {\text{ cosh}}^{{2}} {(}\mu_{{\text{i/e}}} {) + }\chi_{{\text{i/e}}} {\text{ cosh(}}\mu_{{\text{i/e}}} {) + }\eta_{{\text{i/e}}} { = 0,} $$

where *μ*_*i/e*_ = *iκL* is the propagation constant, κ is the wavenumber and *L* is the distance between the supports. Equation (5) is the dispersion relation wavenumber – frequency of a periodic damped pipeline. More specifically, the terms *ψ*_*i/e*_, *χ*_*i/e*_ and *η*_*i/e*_ are functions of Ω_i/e_ and read,

6 $$ \Omega_{{\text{i}}} { = }\frac{{\rho {\text{A}}\omega^{{2}} }}{{{\text{EJ + i}}\omega {\text{ C}}_{{\text{i}}} }}{,} $$

7 $$ \Omega_{{\text{e}}} { = }\frac{{\rho {\text{A}}\omega^{{2}} { } - {\text{ i}}\omega {\text{C}}_{{\text{e}}} }}{{{\text{EJ}}}}. $$

where ω defines the circular frequency. Full derivation of (5) is provided in the Supplementary information: Appendix A.

About the PPR_r_, a dispersion relation between *μ* and *ω* is available in literature^15^ and reads,

8 $$ \cosh (\mu_{i/e} ) = \cosh (i\kappa L) = - \frac{{\cot \left( {\Omega_{i/e} L} \right)\; - \coth \left( {\Omega_{i/e} {\text{L}}} \right)}}{{{\text{csch}} \left( {\Omega_{i/e} L} \right) - \csc \left( {\Omega_{i/e} {\text{L}}} \right)}} $$

Two-dimensional FEMs of the PPR_f_ and PPR_r_ have been modelled via Ansys APDL 19.0 with Euler–Bernoulli beam elements. A time-harmonic rotation *ϕ*_*i/p*_* e*^*i2πft*^ was imposed as input at the left-end of the pipe, and the steady state response *ϕ*_*o/p*_ (*f*) was read as the output rotation at the right-end. The system response in the frequency domain is evaluated as follows,

9 $$ {\text{FRF = 20 log}}_{{{10}}} \left| {\frac{{\phi_{{\text{o/p}}} {\text{(f) }}}}{{\phi_{{\text{i/p}}} {\text{(f)}}}}} \right|, $$

where the *FRF* is the frequency response function expressed in dB. To foster the dynamics of a finite periodic system, the FEM consists of a 40 spans beam.

### Optimization of the stochastic dynamic performance of the VIS

**Figure 3:**
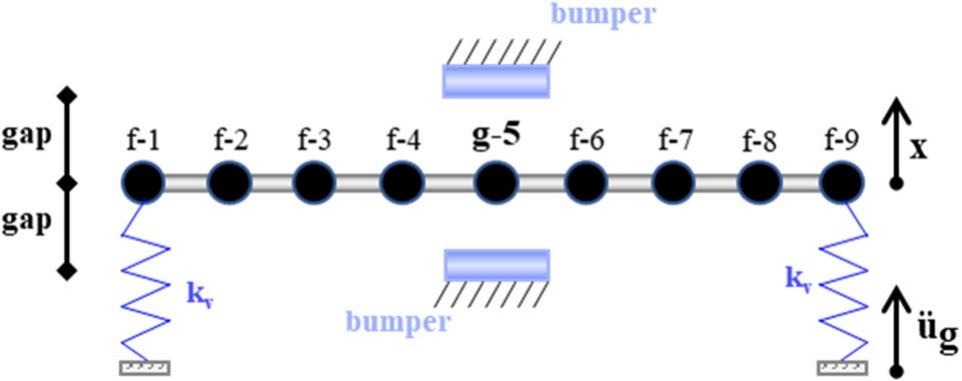
Single span (SS) discretized into 9 nodes that vibrate vertically and rotate; f indicates the nodes that do not experience the impact, whereas g-5 has a two-sided displacement constraint.

To analyse the performance of the VIS, nonlinear transient analyses were carried out for the equations of motion of a single unit cell of the PPR_f_, i.e., a SS beam supported by flexible springs. The aforementioned FEM was adopted to obtain the consistent mass matrix **M** and the symmetric stiffness matrix **K**, whereas the damping matrix **C** was computed with the proportional Rayleigh damping model. The discretized model of the SS equipped with a vibro-impact device is depicted in Fig. 3.

The interface of the SS has a two-sided displacement constraint, such that the node **g-5** impacts two rigid bumpers. In this case, we directly model the VIS and let the system of equations of motion to remain fully linear. The coefficient of restitution (COR) is defined according to Newton’s law^26^:

10 $$ {\text{COR = }} - \frac{{{\text{v}}_{r}^{ + } }}{{{\text{v}}_{r}^{ - } }}{,} $$

where v_r_ is the relative velocity of two colliding bodies, and + and − mean post-impact and pre-impact, respectively. Since the bumpers are fixed to the ground, relative velocities coincide with absolute velocities. Note that Eq. (10) does not need further nonlinearities to be included. However, to take into account the typical limitations of the aforementioned instantaneous model^26,34–36^, very small time steps of the order of 10^–5^ have been used; thus, the simulations showed a limited dependence from time steps^36^.

The system of equations of motion read,

11 $$ {\mathbf{M\ddot{x}}} { + }{\mathbf{C\ddot{x}}}{ + }{\mathbf{Kx}}{ = } - { }{\mathbf{Mr\ddot{u}}}_{{\mathbf{g}}} , $$

where **M**, **C** and **K** are respectively the mass, damping and stiffness matrices, **r** is the influence vector and u_g_ is the ground displacement; the dot represents the derivative wrt the time. When impact occurs, the *g* node is subjected to the impact forces, and the *f* nodes vibrate according to inertial and restoring forces. The equations of motion now read,

12 $$ \left[ {\begin{array}{*{20}c} {{\mathbf{M}}_{{{\text{ff}}}} } & {{\mathbf{M}}_{{{\text{fg}}}} } \\ {{\mathbf{M}}_{{{\text{gf}}}} } & {{\mathbf{M}}_{{{\text{gg}}}} } \\ \end{array} } \right]\left\{ {\begin{array}{*{20}c} {{\mathbf{\ddot{x}}}_{{\text{f}}} } \\ {{\mathbf{\ddot{x}}}_{{\text{g}}} } \\ \end{array} } \right\}{ + }\left[ {\begin{array}{*{20}c} {{\mathbf{C}}_{{{\text{ff}}}} } & {{\mathbf{C}}_{{{\text{fg}}}} } \\ {{\mathbf{C}}_{{{\text{gf}}}} } & {{\mathbf{C}}_{{{\text{gg}}}} } \\ \end{array} } \right]\left\{ {\begin{array}{*{20}c} {{\dot{\mathbf{x}}}_{{\text{f}}} } \\ {{\dot{\mathbf{x}}}_{{\text{g}}} } \\ \end{array} } \right\}{ + }\left[ {\begin{array}{*{20}c} {{\mathbf{K}}_{{{\text{ff}}}} } & {{\mathbf{K}}_{{{\text{fg}}}} } \\ {{\mathbf{K}}_{{{\text{gf}}}} } & {{\mathbf{K}}_{{{\text{gg}}}} } \\ \end{array} } \right]\left\{ {\begin{array}{*{20}c} {{\mathbf{x}}_{{\text{f}}} } \\ {{\mathbf{x}}_{{\text{g}}} } \\ \end{array} } \right\}{ = - }\left[ {\begin{array}{*{20}c} {{\mathbf{M}}_{{{\text{ff}}}} } & {{\mathbf{M}}_{{{\text{fg}}}} } \\ {{\mathbf{M}}_{{{\text{gf}}}} } & {{\mathbf{M}}_{{{\text{gg}}}} } \\ \end{array} } \right]\left\{ {\begin{array}{*{20}c} {{\mathbf{r}}_{{\text{f}}} } \\ {{\mathbf{r}}_{{\text{g}}} } \\ \end{array} } \right\}{\mathbf{\ddot{u}}}_{{\text{g}}} { + }\left\{ {\begin{array}{*{20}c} {0} \\ {{\mathbf{R}}_{{\text{g}}} } \\ \end{array} } \right\}, $$

where **R**_g_ is the impact force vector. Equation (12) is divided in two different equations, one for the non-impacting nodes *f* and one for the impacting node *g*. Since the impact force is unknown, we consider the first equation of the system in (12), as

13 $$ {\mathbf{M}}_{{{\text{ff}}}} {\mathbf{\ddot{x}}}_{{\text{f}}} { + }{\mathbf{C}}_{{{\text{ff}}}} {\dot{\mathbf{x}}}_{{\text{f}}} { + }{\mathbf{K}}_{{{\text{ff}}}} {\mathbf{x}}_{{\text{f}}} { = } - \left( {\left[ {\begin{array}{*{20}c} {{\mathbf{M}}_{{{\text{ff}}}} } & {{\mathbf{M}}_{{{\text{fg}}}} } \\ \end{array} } \right]\left\{ {\begin{array}{*{20}c} {{\text{r}}_{{\text{f}}} } \\ {{\text{r}}_{{\text{g}}} } \\ \end{array} } \right\}{\mathbf{\ddot{u}}}_{{\text{g}}} { + }{\mathbf{M}}_{{{\text{fg}}}} {\mathbf{\ddot{x}}}_{{\text{g}}} { + }{\mathbf{C}}_{{{\text{fg}}}} {\dot{\mathbf{x}}}_{{\text{g}}} { + }{\mathbf{K}}_{{{\text{fg}}}} {\mathbf{x}}_{{\text{g}}} } \right) $$

**Figure 4:**
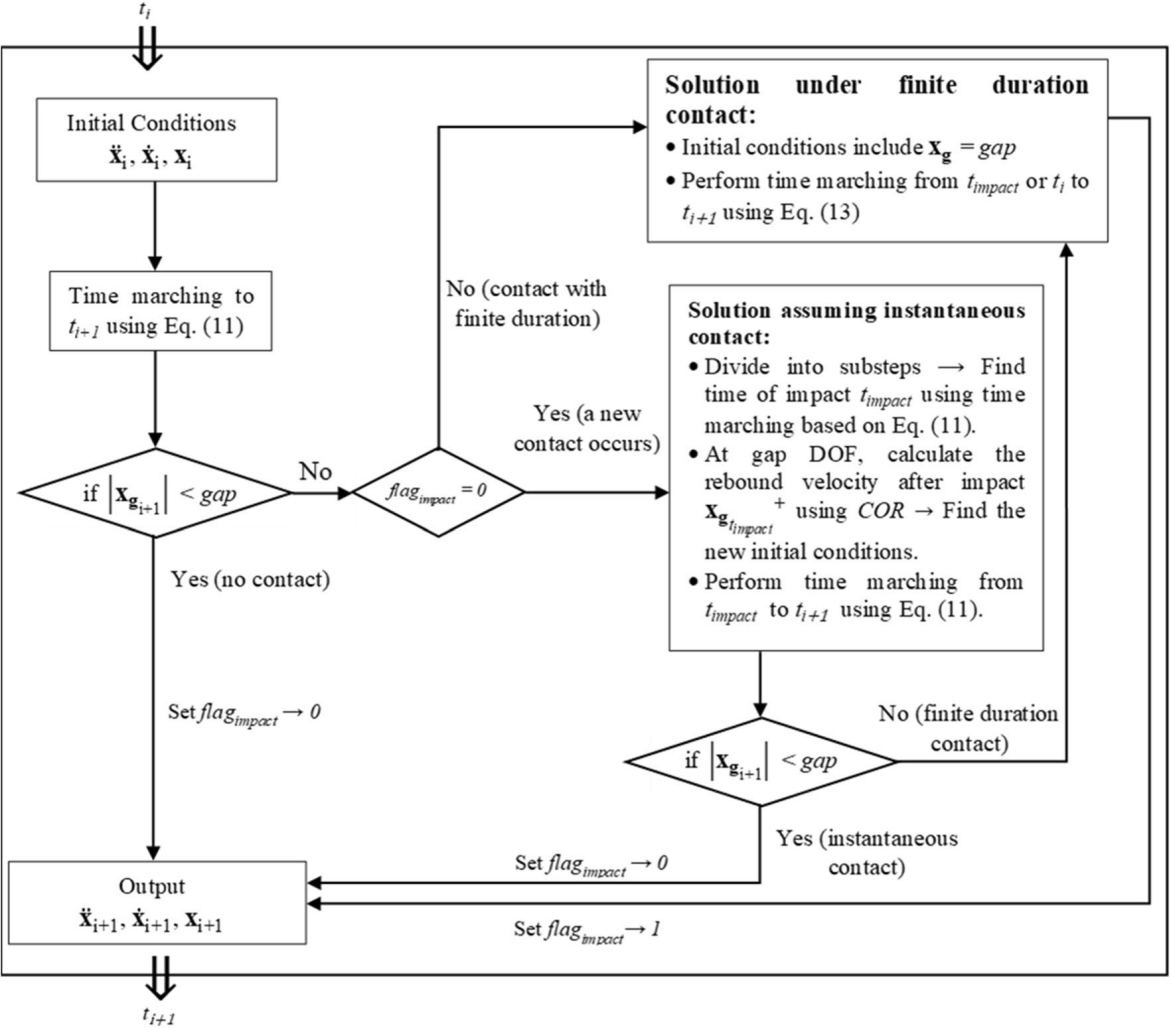
Flow chart for the implementation of the impact in the transient analyses in MATLAB.

To obtain the structural response at the *i*th step of the algorithm, Eq. (12) is employed when the pipe and the bumper are not in contact. Conversely, if the contact occurs, (13) is used. A flowchart that describes the algorithm implemented in MATLAB for the nonlinear impact model is shown in Fig. 4.

At a certain instant $t_{d}$ the energy dissipated by linear viscous damping is computed as follows:

14 $$ {\text{E}}_{{{\text{dam}}}} \left( {{\text{t}}_{{\text{d}}} } \right){ = }\mathop \sum \limits_{{\text{t = 0}}}^{{{\text{t = t}}_{{\text{d}}} }} {\mathbf{dx}}_{{\text{t}}}^{{\text{T}}} {\text{.C}}{.}{\dot{\mathbf{x}}}_{{\mathbf{t}}} , $$

whilst the energy dissipated by the impacts reads,

15 $$ {\text{E}}_{{{\text{imp}}}} \left( {{\text{t}}_{{\text{d}}} } \right){ = }\frac{{1}}{{2}}{ }\mathop \sum \limits_{{\text{i = 0}}}^{{{\text{n}}_{{\text{d}}} }} \left( {{\dot{\mathbf{x}}}^{{\text{ + T}}} {\mathbf{M\dot{x}}}^{{ + }} - { }{\dot{\mathbf{x}}}^{{ - {\text{ T}}}} {\mathbf{M\dot{x}}}^{{ - { }}} } \right){.} $$

where *n*_*d*_ is the number of impacts occurred up to *t*_*d*_, and ${\dot{\mathbf{x}}}^{ - }$ and ${\dot{\mathbf{x}}}^{ + }$ represent the velocity vector before and after impact, respectively. Once the seismic event is extinguished, free decay oscillation occurs in the structure up to a certain time *t*_*r*_ at which the rest condition is fully restored, and the number of impacts is *n*_*im*_. The total input seismic energy reads:

16 $$ {\text{E}}_{{{\text{tot}}}} \left( {{\text{t}}_{{\text{r}}} } \right){\text{ = E}}_{{{\text{dam}}}} \left( {{\text{t}}_{{\text{r}}} } \right){\text{ + E}}_{{{\text{imp}}}} \left( {{\text{t}}_{{\text{r}}} } \right). $$

**Figure 5:**
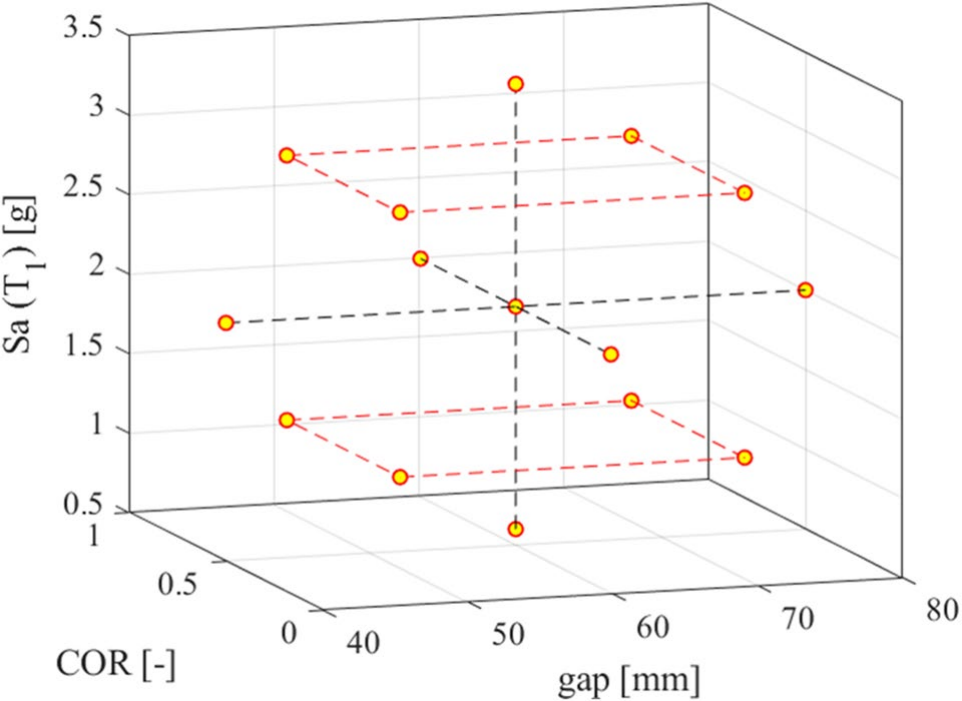
Central composite design (CCD) points for the three factors: gap, COR, and Sa(T_1_).

Therefore, at time instant *t*_*r*_, all the input seismic energy has been dissipated by means of viscous damping and impacts. In these conditions, the design parameters *COR* and *gap* that optimize the seismic performance of the VIS are sought by a multi-objective optimization problem based on two objective functions defined as follows,

17 $$ {\text{O}}_{{1}} \left( {{\text{t}}_{{\text{r}}} } \right){ = }\frac{{{\text{E}}_{{{\text{imp}}}} }}{{{\text{E}}_{{{\text{tot}}}} }}, $$

18 $$ {\text{O}}_{{2}} \left( {{\text{t}}_{{\text{r}}} } \right){\text{ = n}}_{{{\text{im}}}} , $$

where *n*_*im*_ is the number of impacts, *E*_*imp*_ and *E*_*tot*_ are defined by (15) and (16), respectively. The purpose of installing bumpers next to the pipe is to dissipate the largest amount of energy through impacts whilst limiting the number of impacts, to prevent damage to the pipe. Therefore, the objective function *O*_1_ must be maximized (equivalently, − *O*_1_ must be minimized), while *O*_2_ is to be minimized. To reduce the number of transient analyses, we combine CCD and Kriging modelling with the explanatory variables *COR*, *gap*, and *Sa *(*T*_1_), and response variables *O*_1_ and *O*_2_. More precisely, *Sa *(*T*_1_), i.e., the intensity measure (IM)*,* represents the spectral acceleration at the first period *T*_1_ of the system. The 15 yellow points of Fig. 5 indicate the computer-experimental data set-sampling points-defined by the CCD. The ranges of values of the explanatory variables are shown in Table 1.

**Table 1:** Bounds of the CCD variables.

CCD variable	Lower limit	Upper limit
*COR* (−)	0.01	0.99
*gap* (mm)	40	80
*Sa* (*T*_1_) (g)	0.6	3.4

Note that, to take into account the stochastic nature of the seismic input, the IM *Sa*(*T*_1_) has been included in the CCD. The system is nonlinear and sometimes chaotic, see *Supplementary Information—Appendix B*; therefore, a certain variability of the system response wrt the input is expected. As indicated in Table 1, *Sa *(*T*_1_) is bounded by the mean minus/plus the standard deviation of the selected records. Then, to evaluate *O*_1_ and *O*_2_ for the 15 points selected within the CCD, seismic transient analyses are carried out. Each ground motion, see Table 2, is scaled at the relevant value of *Sa*(*T*_1_) and thus, *O*_1_ and *O*_2_ are calculated as the mean of the values obtained by employing all the seismic records described hereinafter. After running all the 15 × 12 analyses needed for CCD, a Gaussian polynomial regression between the 15 mean values by means of the Kriging metamodeling provided by UqLab^43^ has been performed. It has been assumed that the model output is a realization of a Gaussian process defined as the joint distribution of the prediction and the true model response^43^.

**Table 2:** Main characteristics of the selected records.

Event	Country	R, distance (km)	M, magnitude
1. Victoria Mexico	Mexico	13.8	6.33
2. Loma Prieta	USA	3.85	6.93
3. Northridge‐01*	USA	20.11	6.69
4. Montenegro*	Montenegro	25.00	6.90
5. Erzincan	Turkey	13.00	6.60
6. South Iceland*	Island	7.00	6.50
7. L'Aquila Mainshock*	Italy	4.87	6.30
8. Loma Prieta	USA	11.03	6.93
9. Landers*	USA	11.03	7.28
10. South Iceland*	Island	11.00	6.40
11. L'Aquila Mainshock*	Italy	4.63	6.30
12. L'Aquila Mainshock*	Italy	4.39	6.30

Note that the * indicates the records that are employed for performing the incremental dynamic analyses (IDA). The number of impacts, indeed, is related to the maximum velocity exhibited by the system during the transient analysis, and to observe a sufficient number of impacts, the duration of the strong motion must exceed about 40 s.

Then, the Pareto front is used to provide the optimal values of the multi-objective optimization problem by seeking the nondominated solutions among the *O*_1_ and *O*_2_ quantities of (17) and (18) evaluated through the Kriging metamodel. To each nondominated solution of the Pareto front will correspond an optimal triplet (*Sa*(*T*_1_)*, gap, COR*). Therefore, the optimal values of *gap* and *COR* are connected to a certain value of *Sa*(*T*_1_); nonetheless *Sa*(*T*_1_) is not a true design parameter since it characterizes a (seismic) stochastic process.

### Seismic input, fragility assessment, and optimal design parameters selection

**Figure 6:**
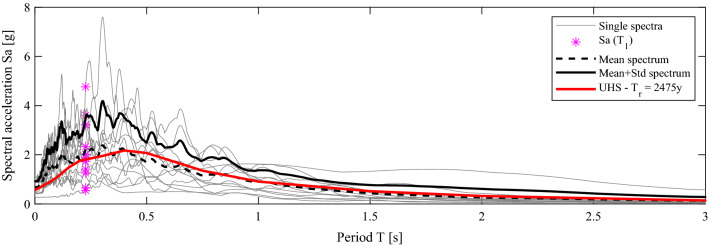
Response spectrum, mean spectrum and mean + standard deviation spectrum matching the UHS; in gray, each individual spectrum of Table 2.

To evaluate the performance of the SS depicted in Fig. 3, a set of twelve natural records with a 2% probability of exceedance in 50 years, are employed; that is, relevant to safe shutdown events (SSE). The selection of the natural seismic records follows the principle sketched in Fig. 6.

Both the mean spectrum and the mean spectrum plus one standard deviation of the selected accelerograms match with the uniform hazard spectrum (UHS) of a specific site, Priolo Gargallo, Sicily in Italy, in a least-square sense. More precisely, let us consider ***s***_**0**_ the target spectrum value vector, that is, the UHS, and evaluate as the spectra matrix of the *n*_*a*_ accelerograms. One can define a vector of *n*_*a*_ selection coefficients, α, where each element can only take a binary value of 1 or 0, and the sum of the elements is equal to *n*_*s*_, i.e., the number of accelerograms to be selected. Thus, the vector **α** that satisfies

19 $$ \min \left( {\left\| {\frac{{{\mathbf{S}}_{{{\varvec{\upalpha}}}} }}{{n_{s} }} - {\mathbf{s}}_{{\mathbf{0}}} } \right\|^{2} } \right) $$

is sought. The selection is performed with all possible combinations of the *n*_*s*_ accelerograms among a set of *n*_*a*_ records. This operation allows to preserve full seismic hazard consistency of the site and minimize the record-to-record variability yet considering the dispersion of the records about the mean spectrum. Table 2 reports the set of accelerograms and their main characteristics.

Finally, we perform the fragility assessment of the SS. Along this line, the fragility function *F*_*DM*_ (*IM*) is defined as the probability that the node **g-5** of Fig. 3 reaches or exceeds some damage measure (DM) for a given ground motion with *IM* = *im*. In particular, the DM is connected to the number of impacts *n*_*im*_, i.e., the engineering demand parameters (EDP), that exceeds a certain threshold. Typically, *F*_*DM*_ (*IM*) is assumed to follow a lognormal distribution, and hence reads,

20 $$ \begin{aligned} {\text{F}}_{{{\text{DM}}}} {\text{(IM) }} & \equiv {\text{ P}}\left[ {\text{EDP > edp|IM = im}} \right]{,} \\ {\text{F}}_{{{\text{DM}}}} {\text{(IM)}} & { = }\Phi \left( {\frac{{{\text{ln(im/X}}_{{\text{m}}} {)}}}{\beta }} \right){,} \\ \end{aligned} $$

where *Φ* is the Gaussian cumulative distribution with a median *X*_*m*_ and a logarithmic standard deviation *β*, calculated as,

21 $$ {\text{X}}_{{\text{m}}} {\text{ = exp}}\left( {\frac{{1}}{{\text{M}}}\sum\limits_{{\text{i}}}^{{\text{M}}} {{\text{ln(IM}}_{{\text{i}}} {)}} } \right){,} $$

22 $$ \beta { = }\left( {\frac{{1}}{{{\text{M}} - {1}}}\sum\limits_{{\text{i}}}^{{\text{M}}} {\left[ {{\text{ln(IM}}_{{\text{i}}} {\text{/X}}_{{\text{m}}} {)}} \right]^{{2}} } } \right)^{{1/2}} {,} $$

where *M* is the number of ground motions considered, and *IM*_*i*_ is the *IM* value associated with onset of DM for the ith ground motion. The values of *IM*_*i*_ are the results of the IDA performed on the SS. Commonly, the IDA involves scaling each ground motion in a suite until it causes the exceedance of some DM^52^. However, for the purpose of generality, *F*_*DM*_ (*IM*) in (20) is calculated for all the feasible *n*_*im*_ = *EDP* corresponding to the Pareto front. In fact, the Pareto front highlights the optimal design parameters *gap* and *COR*. Therefore, each optimal value of the Pareto front corresponds to a fragility surface (*F*_*DM*_ (*IM*), *IM*, *EDP*). The corresponding volume V_j_ under the jth surface is computed as follows

23 $$ {\text{V}}_{{\text{j}}} = \int\limits_{IM} {\int\limits_{EDP} {{\text{F}}_{{{\text{DM}}}} {\text{(IM) }}dIM \, dEDP} } , $$

where j indicates the jth optimal solution of the Pareto front. According to the law of total probability, (23) is proportional to the total probability of exceedance of all the *edp*s. Then, one should perform the fragility assessment for all the *j* = {1,…,n} Pareto front solutions and select the optimal couple gap—COR that minimizes (23). In this work, an application of this procedure is shown for three optimal couples.

## Results

### Dispersion* curves* of the* PPRs* and enhancement of the* vibration mitigation*

The pipe cross section has an outer and inner diameter of 0.2731 m and 0.2639 m, respectively, whilst the inertia *J* of the cross section is 3.4977e−5 m^4^. The span length *L* is 12 m, and the modulus of elasticity *E* and the mass density *ρ* are 200 GPa and 7800 kg/m^3^, respectively. The stiffness *k*_*v*_ of the pipe rack’s pillars reads 17.9 MN/m and the mass *m*_*pr*_ of the repetitive unit of the coupled system PPR is equal to 22,880 kg. Consequently, the first natural frequency of the PPR_f_ reads *ω*_*pr,1*_ = (*k*_*v*_*/m*_*pr*_)^0.5^ = 4.45 Hz.

**Figure 7:**
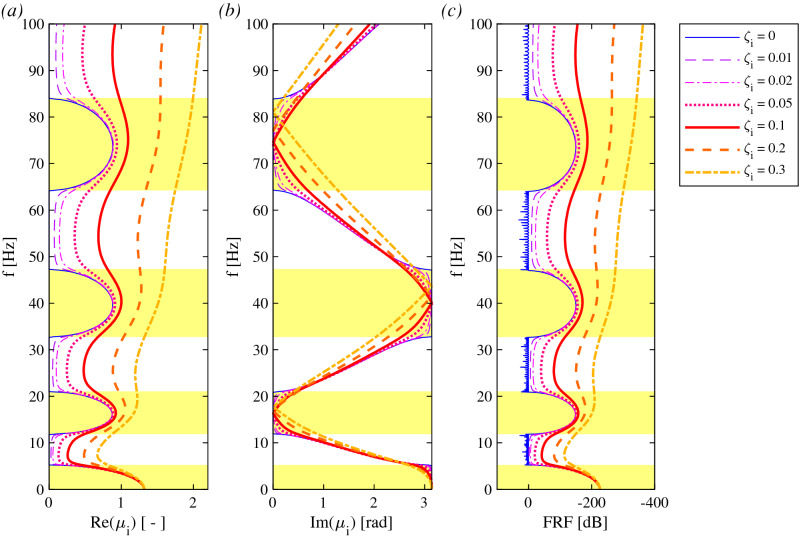
Dispersion curves for an internally damped PPR_r_ as (**a**) function of the real part of μ_i_, (**b**) function of the imaginary part of μ_i_; (**c**) numerical FRF of the finite periodic (40 spans) FEM. The bandgaps are indicated in yellow.

When the pillars’ stiffness is considerably higher than the pipe’s flexural stiffness, the pillars act as rigid supports wrt the flexural behaviour of the pipe. Thus, the PPR_r_ is modelled as a beam supported by simple supports. The dispersion diagrams of the internally damped PPR_r_, are calculated via Eq. (6) and are depicted in Fig. 7.

Figure 7 shows the dispersion diagrams (*μ−f*) for seven values of material damping *ζ*_*i*_ of Eq. (3). The yellow bands in Fig. 7a, b highlight four bandgaps in the following frequency ranges: [0–5.25] Hz, [11.94–21.01] Hz, [32.79–47.27] Hz and [64.30–84.03] Hz. The dispersion diagrams of Fig. 7 reveal common properties of linear periodic systems. The blue curve represents the undamped pipe and clearly defines the bandgaps in the regions where *Re*(*μ*)* ≠ 0* and, consequently, *Im*(*μ*) = *0* or *Im*(*μ*) = *π*, unveiling the existence of pure evanescent waves. Instead, for damped pipes with nonzero values of *ζ*, *Re*(*μ*)* ≠ 0* in the overall frequency domain, since attenuating oscillatory waves can always be observed for all frequencies. However, the damping is included in the main structure, and clearly enlarges the attenuation rate *Re*(*μ*). This effect is more evident as the frequency range increases. Figure 7c shows the *FRF* of (9), evaluated with the FE software. A good agreement is observed between Fig. 7c and the dispersion curves of Fig. 7a, b, in terms of passbands, bandgaps and attenuation rates.

**Figure 8:**
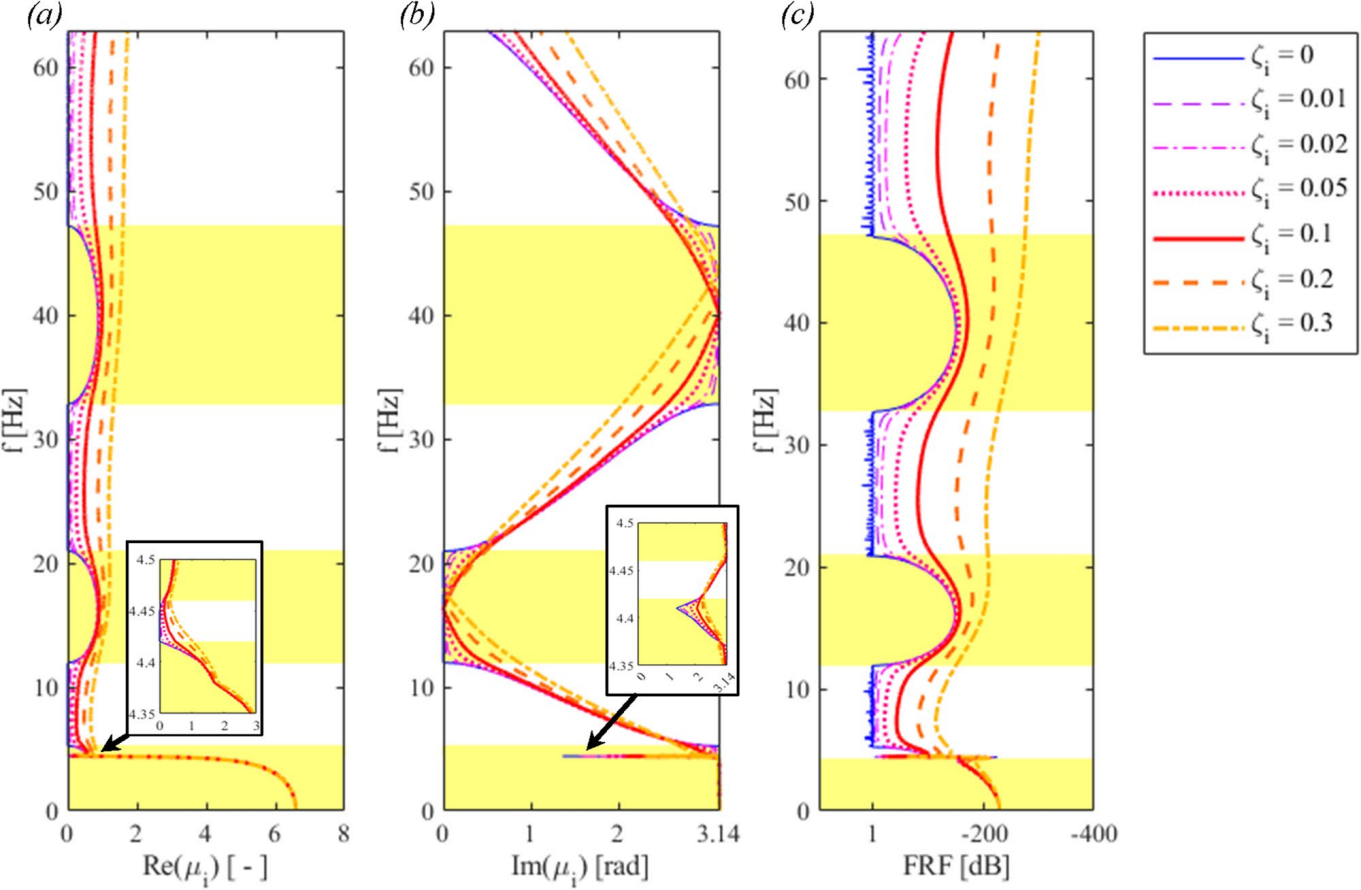
Dispersion curves for an internally damped PPR_f_: (**a**) real part of μ_i_, (**b**) imaginary part of μ_i_; (**c**) the numerical FRF.

Let us now consider the PPR_f_ depicted in Fig. 2b. The pipe rack is modelled by a spring-mass oscillator that matches the first lateral mode of the rack. Iqbal et al.^17^ demonstrated the effectiveness of such an approximation. The relevant dispersion curves are calculated via (5) and plotted in Fig. 8.

Fig. 8a, b show the dispersion relations of the PPR_f_, where a local resonance is observed around the first natural frequency of the pipe rack *ω*_*pr,1*_ = 4.45 Hz. The first bandgap, that initially ranged from 0 to 5.25 Hz, is now divided into two different bandgaps. Generally, bandgaps can be tuned with local resonances, whereas the Bragg scattering induces bandgaps that are constrained by the periodic system dimensions. Nevertheless, in this paper the PPR_f_’s first natural frequency belongs to the first bandgap frequency range, and therefore no additional bandgaps appear. Conversely, Fig. 8a, b depict a zoom on a narrow passband that opened due to the pipe rack’s resonance. Finally, one can notice a good agreement between the numerical FRF of Fig. 8c and the analytical dispersion diagrams of Fig. 8a, b. Again, the damping induces an attenuation rate that increases with the frequency.

The combination of Eq. (7) with (8) allows for plotting the dispersion diagrams and the bandgaps relevant to the external damping case. Also in that situation, favourable bandgap zones can be obtained; for brevity, they are not shown or commented on.

### Optimization results

**Figure 9:**
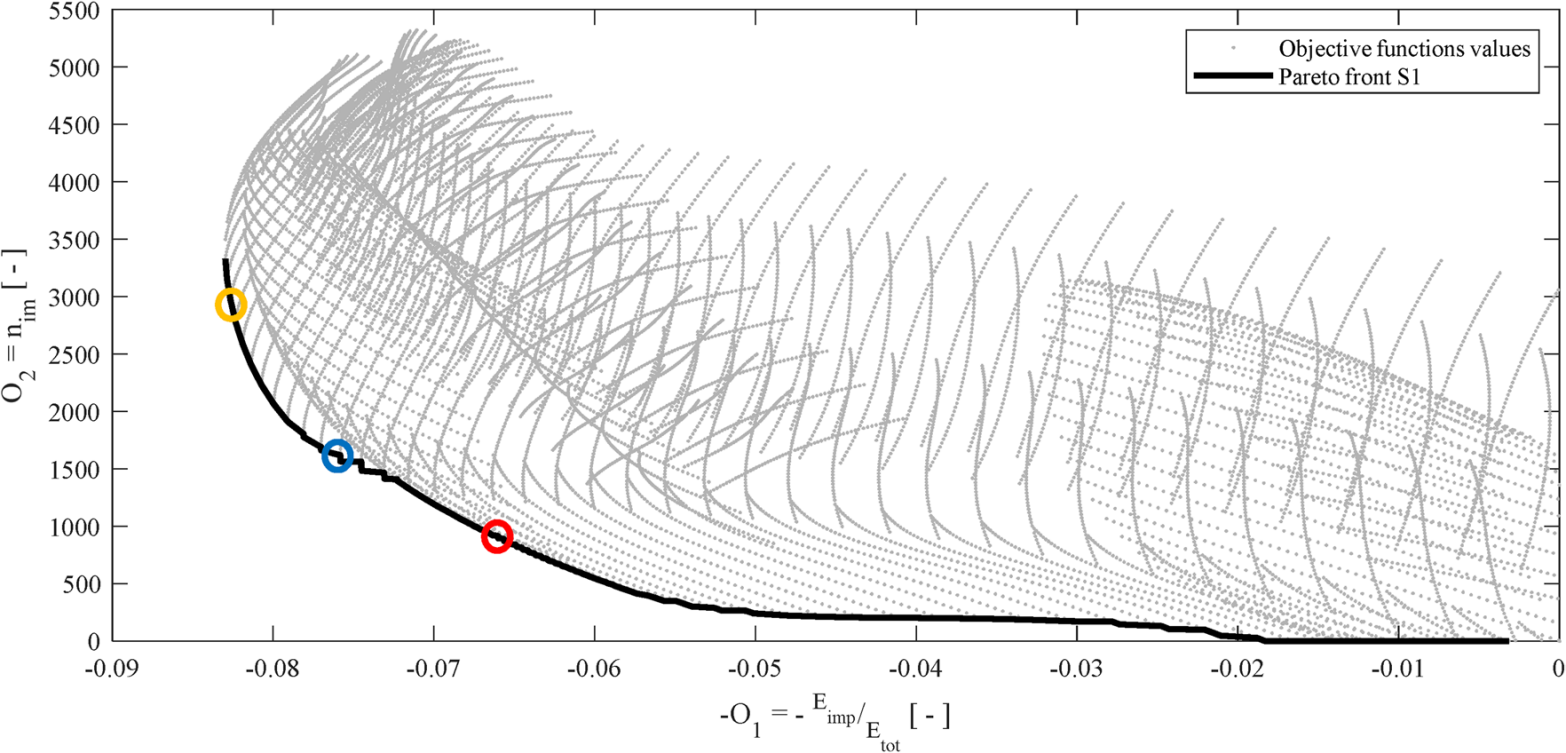
Plot of all the realizations of the Kriging metamodel and the Pareto front **S1**; the circles indicate the three optimal solutions investigated in the Subsection “Fragility assessment and selection of the optimal solution”.

As anticipated in a previous subsection, the Pareto front **S1** depicted in Fig. 9 pinpoints the nondominated solutions among all the possible realizations evaluated with the Kriging model. This Pareto front is called **S1** to avoid confusions with Fig. 12.

**Figure 10:**
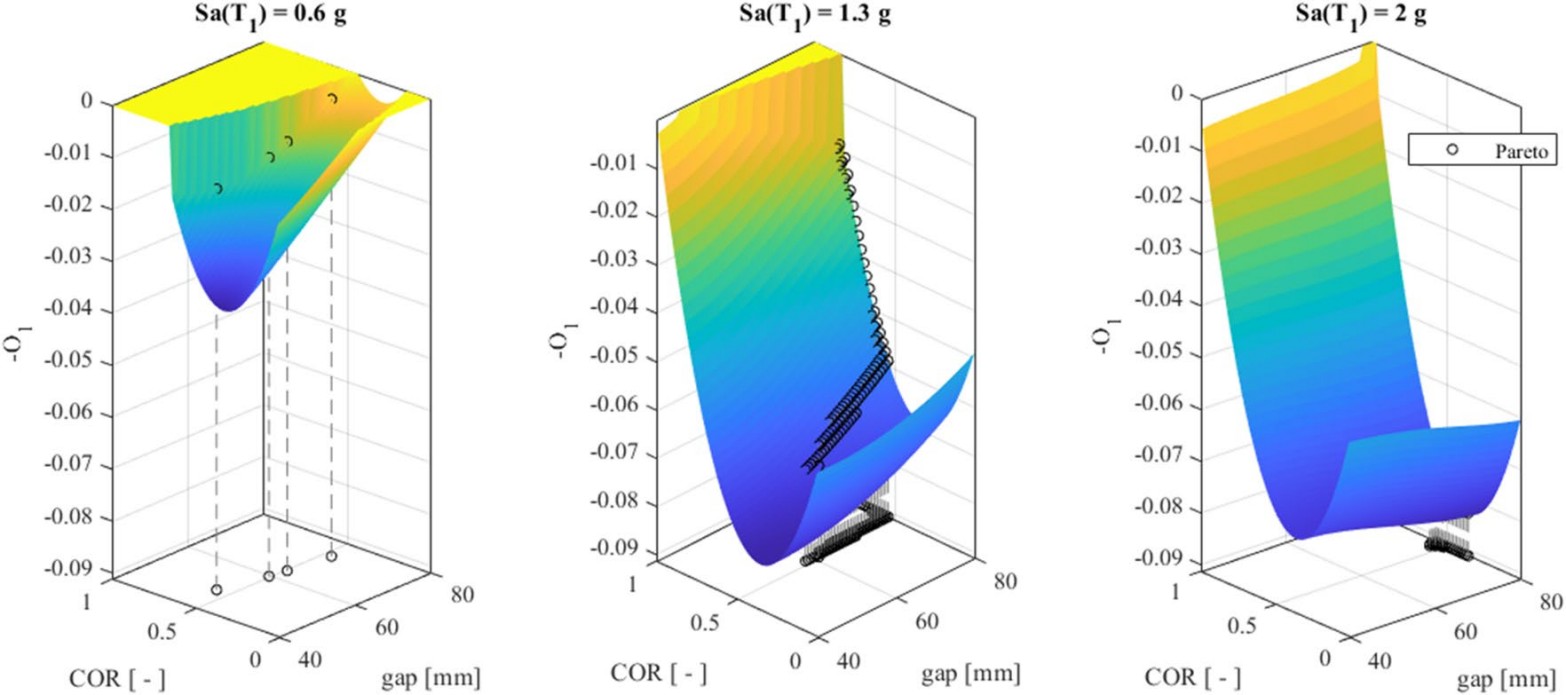
O_1_ surfaces for (**a**) the “optimal” Sa(T1) equal to 0.6 g; (**b**) equal to 1.3 g; and (**c**) 2 g.

The surrogate Kriging metamodel generates *O*_1_ and *O*_2_ as 3D-arrays for the parameters *gap*, *COR* and *Sa*(*T*_1_). Therefore, the Pareto front highlights the optimal triplets (*Sa*(*T*_1_)*, gap, COR*), and the optimal values of *gap* and *COR* corresponds to a certain *optimal* value of *Sa*(*T*_1_). Nonetheless, *Sa*(*T*_1_) is characterized by randomness and influences the optimal solutions. For clarity, the three surfaces that depict the values of *O*_1_ wrt three *optimal Sa*(*T*_1_)*,* equal to 0.6 g, 1.3 g and 2 g, are depicted in Fig. 10.

**Figure 11:**
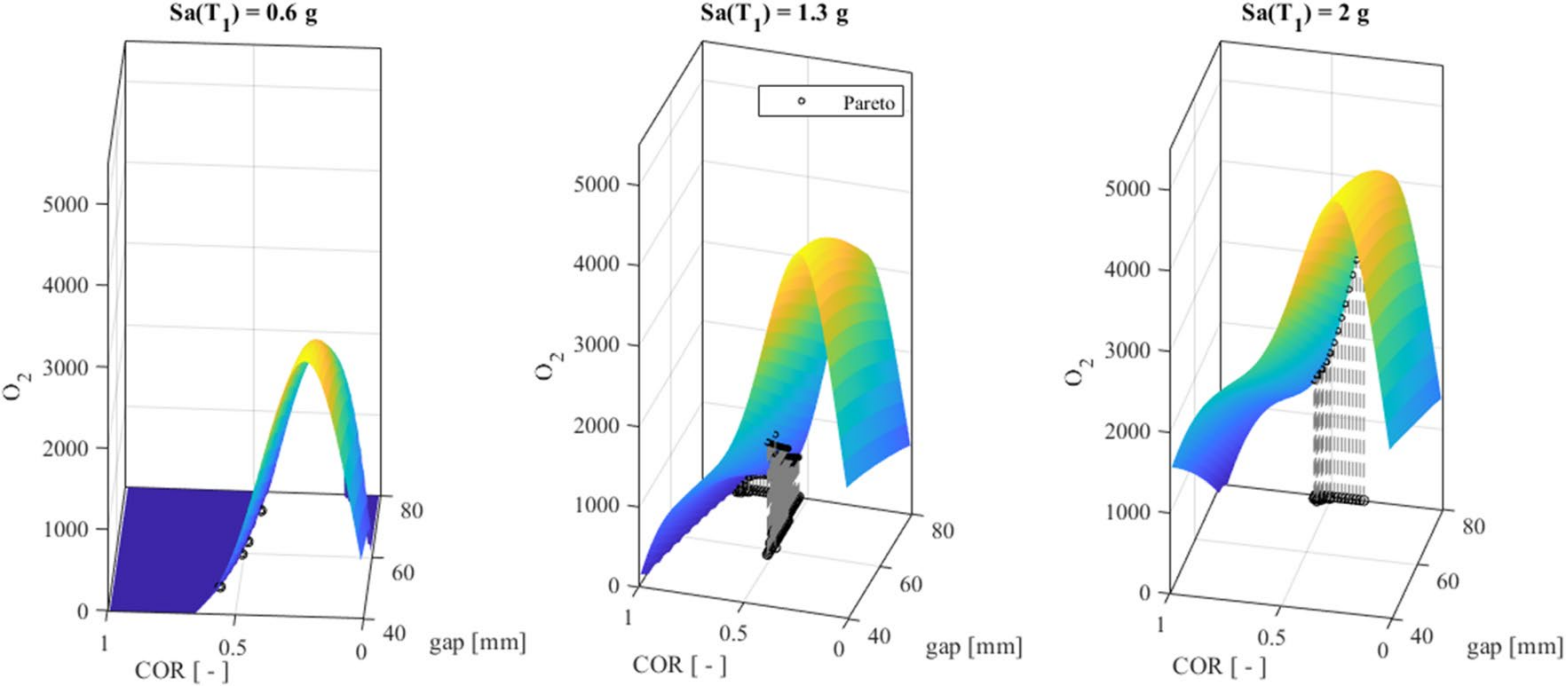
O_2_ surfaces for (**a**) the optimal Sa(T1) equal to 0.6 g, (**b**) 1.3 g, and (**c**) 2 g.

Note that the objective function *O*_1_ defined in (17), must be maximized. Nonetheless, we plot *-O*_1_ in Fig. 10 and then seek its minimum values. The surfaces *O*_2_ of (18) refer to the *optimal Sa*(_1_) equal to 0.6 g, 1.3 g and 2 g in Fig. 11.

The black circles in both Figs. 10 and 11 represent the Pareto front of Fig. 9.

**Figure 12:**
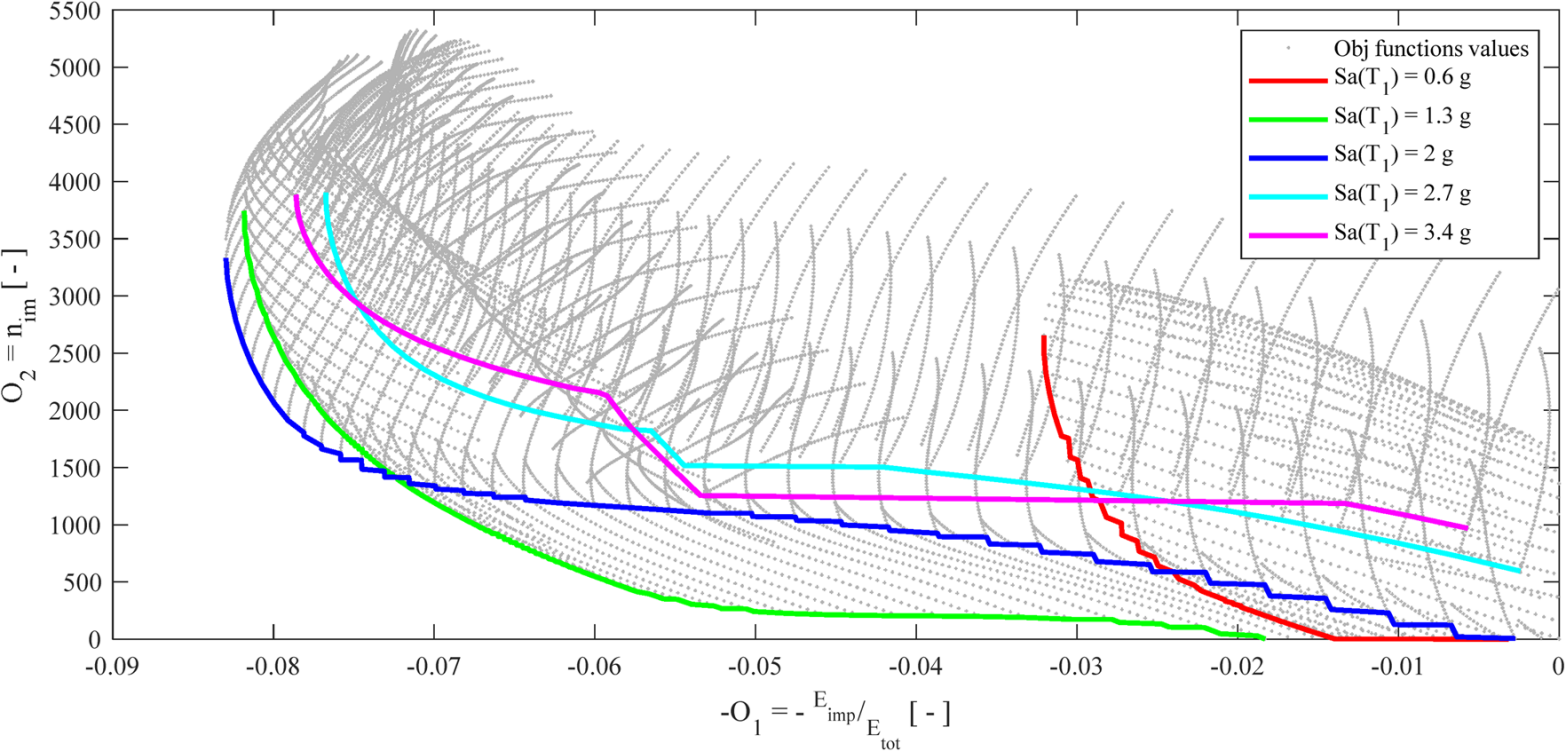
Pareto fronts obtained for certain Sa(T_1_) levels.

To underline the sensitivity of the optimization problem to the seismic input *Sa*(*T*_1_), Fig. 12 shows the Pareto fronts corresponding to several levels of *Sa*(*T*_1_).

**Figure 13:**
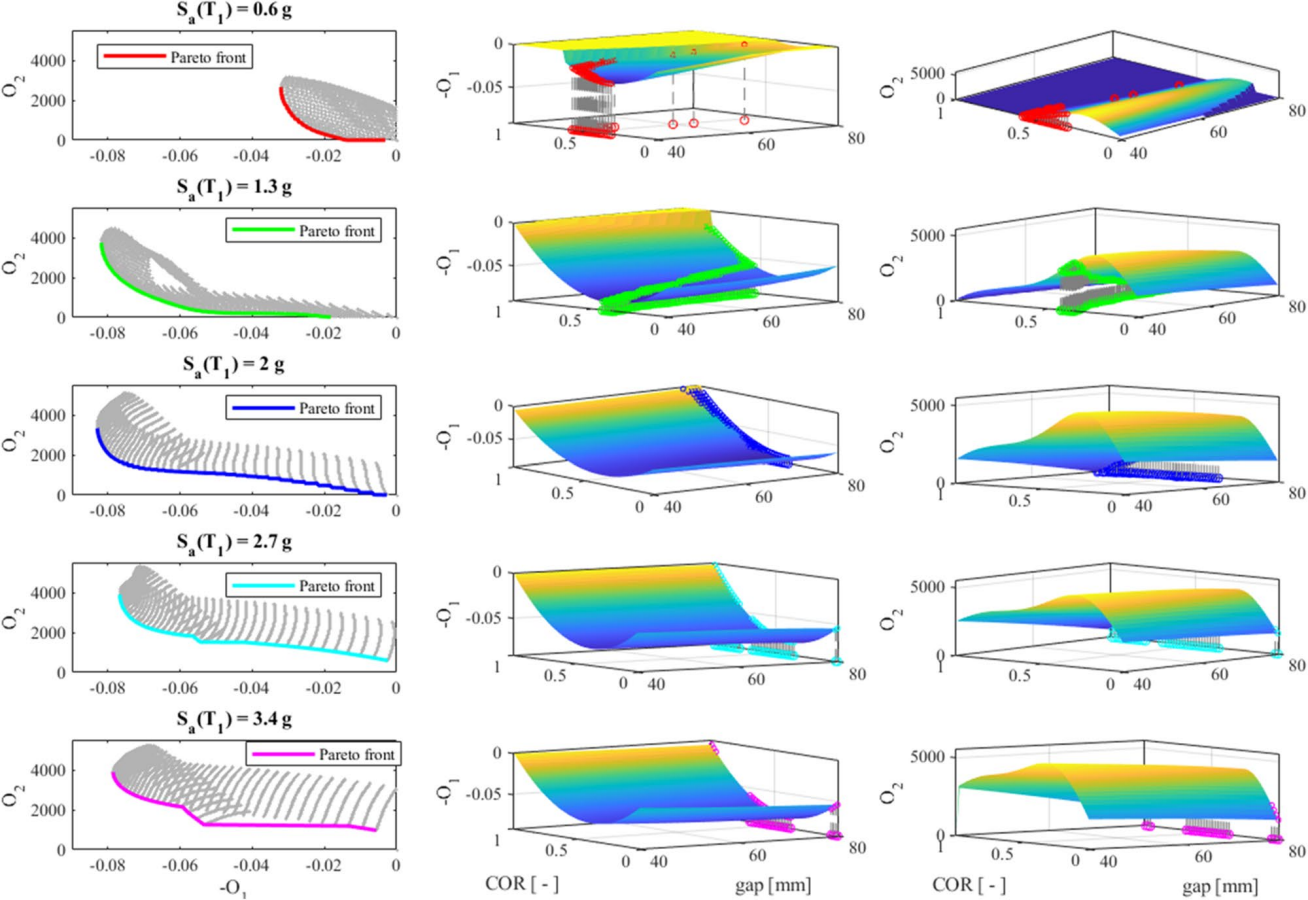
On the left column, each single Pareto front of Fig. 12; on the middle and the right column, the −O_1_ and O_2_ surfaces, respectively.

It is clear that the optimal solutions differ significantly as the severity level of the seismic input varies. For clarity, Fig. 13 depicts all the Pareto fronts of Fig. 12 along with the relevant surfaces *O*_1_ and *O*_2_.

One can clearly note that the optimization is sensitive to the seismic input *Sa*(*T*_1_); therefore, the inclusion of the IM in the CCD is justified. A few more general considerations arise from the optimization results. Both the objective functions *O*_1_ and *O*_2_ depend more on the *COR* than on the *gap*. In particular, optimal values of *COR* are found in the whole range 0–1, whereas large *gap* values are optimal for strong earthquakes, say *Sa*(*T*_1_) equal to 2 g, 2.7 g, and 3.4 g. For lower values of *Sa*(*T*_1_), the optimal *gap*s cover the whole range 40–80 mm.

### Fragility assessment and selection of the optimal solution

**Figure 14:**
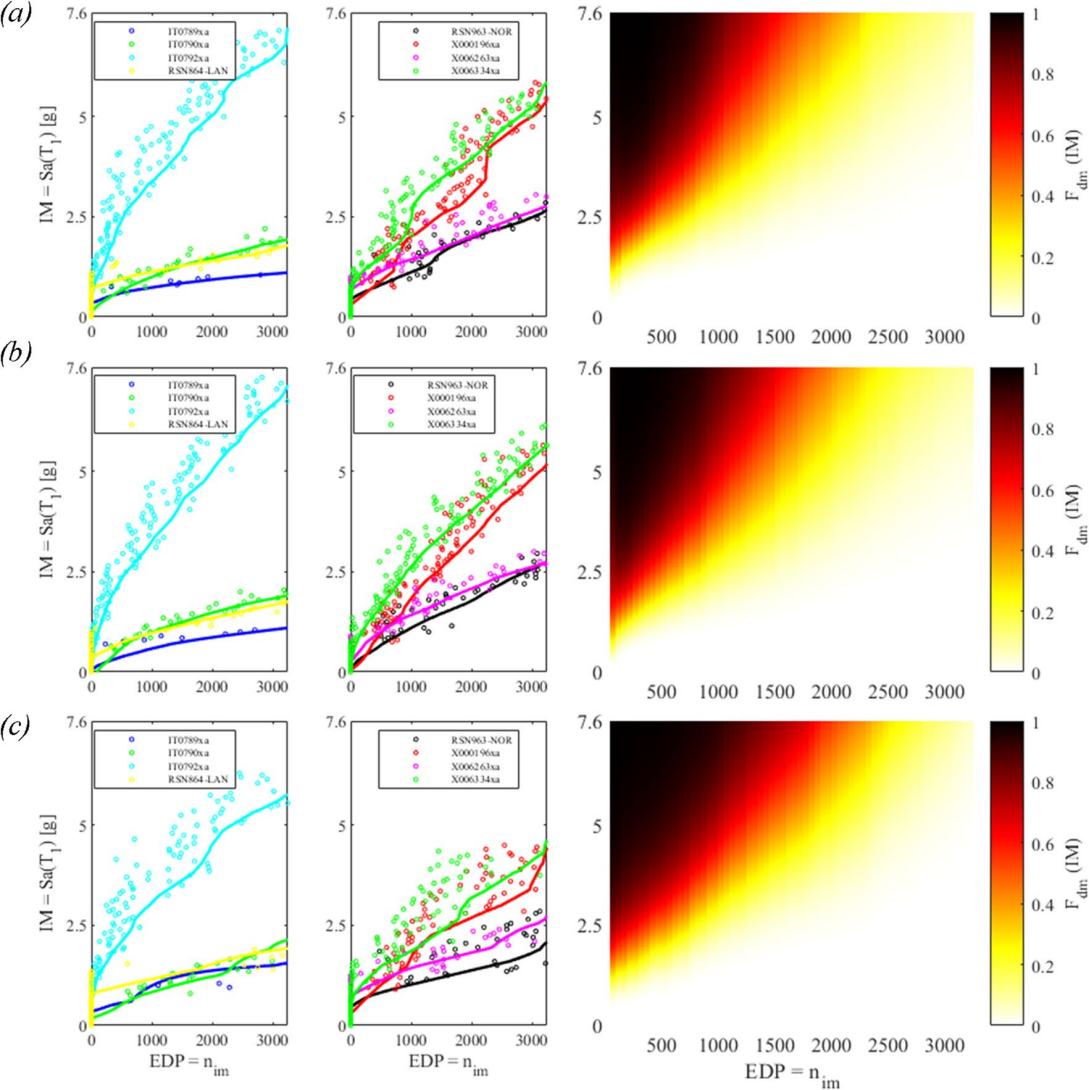
IDA curves on the left side and 2D color plot of the fragility function on the right side for: (**a**) the first optimal point with gap = 64 mm, COR = 0.49; (**b**) the second optimal point with gap = 58 mm, COR = 0.52; and (**c**) the third optimal point with gap = 80 mm, COR = 0.39. These optimal points are indicated by the circles in Fig. 9.

To perform the fragility assessment, three optimal solutions among those of the Pareto front in Fig. 9 are randomly taken; and the IDA are calculated for the eight ground motions indicated with the * in Table 2. Figure 14 reports the results of the IDA and the relevant 2D fragility functions.

**Figure 15:**
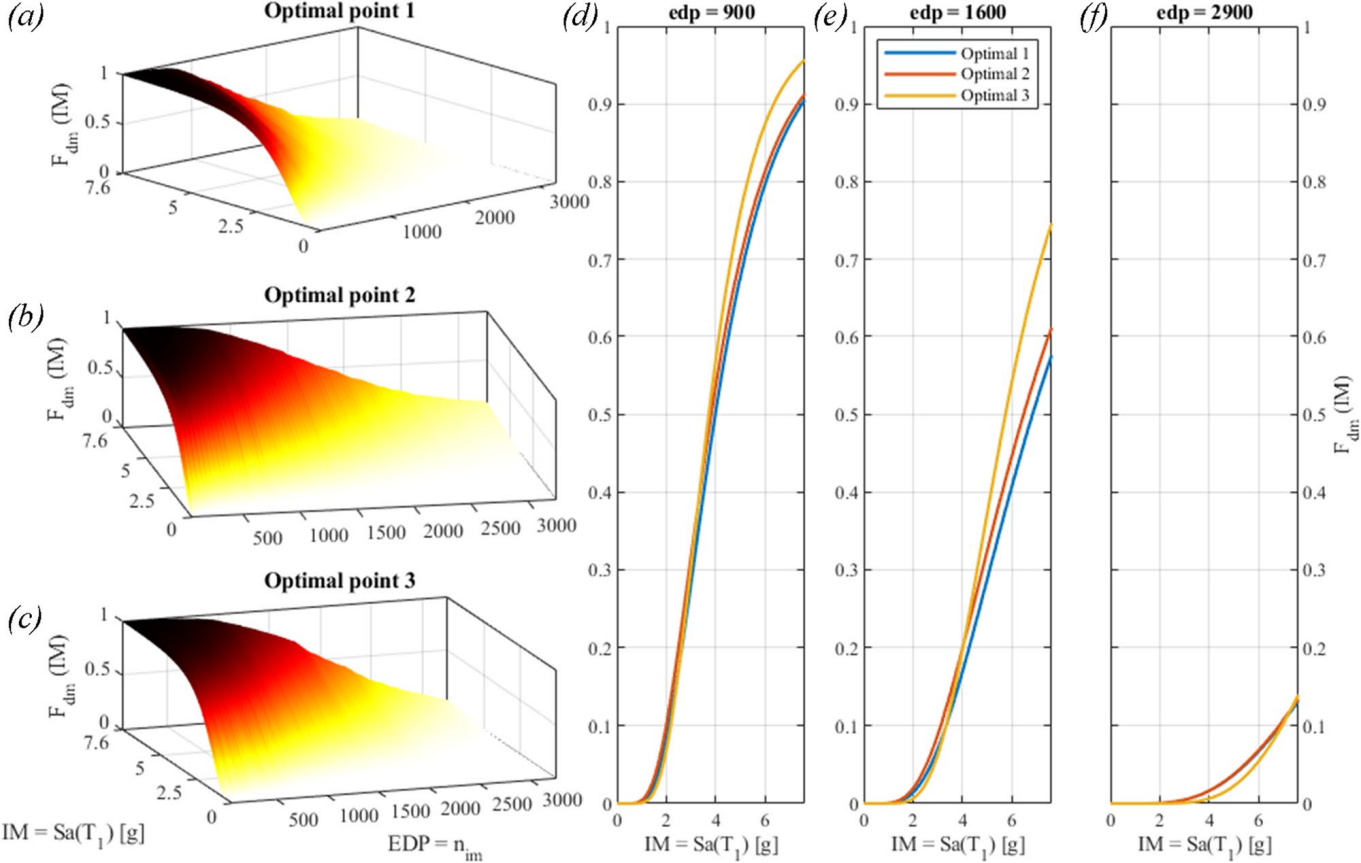
Fragility surfaces for: (**a**) the Optimal solution #1; (**b**) the Optimal solution #2; (**c**) the Optimal solution 3; and relevant fragility curves for: (**d**) n_im_ = 900; (**e**) n_im_ = 1600; (**f**) n_im_ = 2900; these values of n_im_ were taken from the values of O_2_ in Fig. 9.

The impact phenomenon may generate chaotic motion, as described in the *Supplementary Information—Appendix B;* hence the IDAs result in responses that are not unique for a certain *EDP* = *n*_*imp*_. Vamvatsikos et al.^52^ described the occurrence of multiple capacity points, and recommended to handle this ambiguity by an ad hoc, specified procedure, i.e., by conservatively defining the limit state point as the lowest IM. However, we preferred to filter the numerical data of the IDAs, see the circles in Fig. 14, with a moving average technique that resulted in the solid curves of Fig. 14. Indeed, the IDA curves appear rather noisy because of the nonlinear and sometimes chaotic behaviour of the VIS. Then, the fragility surfaces are calculated by Eq. (20) and are plotted both in Figs. 14 and 15.

Moreover, we can compute the volumes underneath the surfaces of Fig. 15a–c with (23). The lower the surface, the lower the probability of exceedance of the *edp*, namely the number of impacts n_imp_. The volumes relevant to Fig. 15a–c read: *V*_1_ = 6698 g, *V*_2_ = 7069 g, and *V*_3_ = 7270 g. Therefore, the Optimal solution #1 is the one that minimizes the volume under the fragility surface.

Finally, and for the sake of completeness, the reader can appreciate the speed of the algorithm sketched in Fig. 4 by using Optimal solution #1. In this respect, Table 3 reports the CPU time required to simulate the response of the optimal VIS to the records listed in Table 2 using MATLAB. The simulations run on a processor with a clock speed of 3.00 GHz and 4 cores.

**Table 3:** CPU time requested for solving the transient analyses for each seismic record of Table 2.

Event	1	2	3	4	5	6	7	8	9	10	11	12
Event duration (s)	100	100	100	24.5	25	25	44	40	48.2	72.5	55	21.3
CPU time (s)	32.1	36.0	36.8	12.1	9.8	10.1	16.8	16.0	18.8	26.6	19.2	9.2

One can observe that the CPU time employed to solve the system of Eq. (11) is effective in relation to the event duration.

## Discussion and outlooks

In this paper, the enhanced attenuation properties of two periodic damped pipelines coupled with pipe racks (PPRs) have been shown; and the relevant results have been confirmed by the FE software Ansys on a 40-spans PPR. Two damping models were proposed in view of vibration mitigation, i.e., internal material damping, and external viscous damping. A local resonance was observed around the first natural frequency of the PPR *ω*_*pr,1*_ = 4.45 Hz. The damping clearly enlarges the attenuation rate *Re*(*μ*), and this effect is more evident as the frequency range increases. As a result of the analyses, we have found a good agreement between the frequency response function (FRF) of the FE models and the analytical band structures.

Then, a generic single span (SS) of the PPR equipped with a vibro-impact device was considered; and due to the nonlinearities, a design optimization procedure was conceived and carried out. The procedure aims to maximize the dissipation energy and to minimize the number of impacts. Both objective functions resulted to be more sensitive to the *COR* than to the *gap*; the response surface was generated by the Kriging metamodel with a Gaussian 2nd order-polynomial regression. It was found that the metamodels were endowed with sharp curvatures wrt to *COR*. Optimal values of *COR* were found in the whole range 0–1; large *gap* values appear to be optimal for strong earthquakes, say *Sa*(*T*_1_) equal to 2 g, 2.7 g, and 3.4 g, whereas optimal *gap*s are found in the whole range 40–80 mm for lower values of *Sa*(*T*_1_). Moreover, we have found that the optimal solution with *gap* = *64* mm and *COR* = 0.49 is the one that minimizes the probability of exceeding the damage states (DM) connected to the engineering demand parameter (EDP), that is, the number of impacts. The incremental dynamic analysis (IDA) resulted in curves that are not unique for a certain *EDP*, i.e., noisy, mainly due to the chaotic behaviour of the VIS. In addition, the IDA curves displayed significant record-to-record variability: for example, in Fig. 14a, the *EDPn*_*im*_ = 3000 was reached for values of *Sa*(*T*_1_) equal to about 1 g, 1.6 g, 1.7 g, 2.4 g, 2.5 g, 5 g, 5.3 g and 7 g. This result, however, must be imputed to the frequency content of the records and the nonlinear response of the vibro-impact that exhibits a strong dependency upon frequency and amplitude of excitation, and sometimes chaos. To investigate this last issue, we have excited the single span (SS) with periodic loadings for a restricted range of frequencies in the vicinity of *ω*_*pr,1*_, and have reported the relevant bifurcation diagrams in the *Supplementary Information – Appendix B*. When impact does not occur, the bifurcation diagram shows that the system is linear and periodic. Conversely, the impact activates higher modes of vibrations, and non-periodic solutions that can be found in the bifurcation diagram. Therefore, three of these trajectories have been investigated, and the largest Lyapunov exponents were found to be higher than zero, thus indicating the presence of divergence and chaos.

In conclusion, we have shown how to evaluate the safest design solution for a nonlinear dissipation system despite the (seismic) stochastic nature of the loading. Nevertheless, some challenges remain undisclosed. The defined dispersion curves concern the linearly damped system without impact-induced dissipation. Nonlinear waves, in fact, distort as they propagate and change their original shape along the periodic medium; therefore, numerical transient analyses of a multiple-span pipe rack with vibro-impact devices may reveal unintended periodic features of the impacting repetitive structure.

## Supplementary Information

Supplementary Information.

## Data Availability

Raw data have been provided in a supplementary file. For more details, please contact the corresponding author.
