# Supplementary material for: Pipe vibration attenuation through internal damping and optimal design of vibro-impact systems
Source: Sci Rep. 2023 Apr 20;13:6510. doi: 10.1038/s41598-023-33640-y (PMC10119187; doi:10.1038/s41598-023-33640-y)
Supplement: Supplementary file 1 — Supplementary Information. [file 41598_2023_33640_MOESM1_ESM.docx]

# Pipe vibration attenuation through internal damping and optimal design of vibro-impact systems

- SUPPLEMENTARY MATERIAL -

Fabrizio Aloschi*^1,2^, Roberto Andreotti^1^, Oreste Salvatore Bursi^1^

^1^University of Trento, Department of Civil, Environmental and Mechanical Engineering, Via Mesiano 77, 38123 Trento, Italy

^2^IGF - Ingenieurgesellschaft Dr. Ing. Fischbach mbH, An der Vogelrute 2, 50374 Erftstadt-Lechenich, Germany

*Corresponding author, fabrizio.aloschi@unitn.it

*Appendix A - Dispersion analysis of periodic damped PPR_f_*

For the PPR_f_ and the internal damping model, Fig. 2 (a) and (c), the Euler-Bernoulli equation reads:

| $\frac{\text{∂}^{2}}{\text{∂}\text{z}^{2}}\left[ \text{EJ}\frac{\text{∂}^{2}\text{w(}\text{z}\text{,t)}}{{\text{∂}\text{z}}^{2}} \right]\text{ + }\frac{\text{∂}^{2}}{\text{∂}\text{z}^{2}}\left[ \text{C}_{\text{i}}\frac{\text{∂}^{2}\frac{\text{∂ w(z,t)}}{\text{∂t}}}{\text{∂z}^{2}} \right]\text{ + }\text{ρA}\frac{\text{∂}^{2}\text{w(}\text{z,t}\text{)}}{\text{∂t}^{2}}\text{ = 0, }$ | *(A.1)* |
| --- | --- |

where *ρ, E, A* and *J* are, respectively, the material density, the Young modulus, the area and the inertia of the beam cross section. The term *w(z,t)* is the beam transversal displacement. *C_i_* is the internal damping coefficient. Instead, for an externally damped PPR_f_, see Fig. 2 (a) and (d), the Euler-Bernoulli equation reads:

| $\frac{\text{∂}^{2}}{\text{∂}\text{z}^{2}}\left[ \text{EJ}\frac{\text{∂}^{2}\text{w(z,t)}}{\text{∂z}^{2}} \right]\text{ }\text{+}\text{ }\text{C}_{\text{e}}\frac{\text{∂ w(z,t)}}{\text{∂t}}\text{ }\text{+}\text{ }\text{ρA}\frac{\text{∂}^{2}\text{w(}\text{z,t}\text{)}}{\text{∂t}^{2}}\text{ }\text{=}\text{ }\text{0,}$ | *(A.2)* |
| --- | --- |

where C_e_ is the external damping coefficient. In (A.1) and (A.2), the damping coefficients read,

| $\text{C}_{\text{i}}\text{ = }\frac{\text{2 }\text{ζ}_{\text{i,n}}\text{ EJ}}{\text{ω}_{\text{n}}}\text{,}$ $\text{C}_{\text{e}}\text{ }\text{= 2 }\text{ζ}_{\text{e}\text{,n}}\text{ }\text{ρ}\text{A }\text{ω}_{\text{n}}\text{,}$ | *(A.3)*  *(A.4)* |
| --- | --- |

where *ζ_i/e,n_* is the modal damping ratio, *i/e* specifies internal or external damping, and *ω_n_* is the modal frequency. The free wave oscillations of the *j^th^* support are expressed as,

| $\text{w}_{\text{j}}\left( \text{z}\text{,t} \right)\text{ = }\text{w}_{0}\text{e}^{\text{j}\text{μ}}\text{e}^{\text{іω}\text{t}}\text{ =}\text{ w}_{\text{0,j}}\text{(z)}\text{e}^{\text{іω}\text{t}}\text{,}$ | (A.5) |
| --- | --- |

where $\mu$ is the propagation constant that is defined as

|  | (A.6) |
| --- | --- |

where *κ* is the wavenumber, *L* is the distance between the supports and *i* is the imaginary unit. From Eqs. (A.1) and (A.2), and by considering Eq. (A.5), the following fourth-order ordinary differential equation can be derived for the case of internal and external damping, respectively:

| $\text{(}\text{EI}\text{ }\text{+ }\text{iω}\text{ }\text{C}_{\text{i}}) \text{w}^{\text{IV}}\left( \text{z} \right) \text{- ρ}\text{ }\text{A}\text{ }\text{ω}^{2}\text{ }\text{w}\left( \text{z} \right)\text{ = 0,}$ | (A.7) |
| --- | --- |
| $\text{EI}\text{ }\text{w}^{\text{IV}}\left( \text{z} \right)\text{ + }\text{(}\text{i}\text{ω}\text{ }\text{C}_{\text{e}} \text{- }\text{ρ}\text{ }\text{A}\text{ }\text{ω}^{2}\text{) }\text{w}\left( \text{z} \right)\text{ = 0}.$ | (A.8) |

Both the equations can be rewritten in a compact form:

| $\text{w}^{\text{IV}}\left( \text{z} \right)\text{ = }{\text{Ω}_{\text{i}}}^{4}\text{ w}\left( \text{z} \right)\text{,}$ | (A.9) |
| --- | --- |
| $\text{w}^{\text{IV}}\left( \text{z} \right)\text{ =}{\text{ Ω}_{\text{e}}}^{4}\text{ w}\left( \text{z} \right)$, | (A.10) |

where Ω_i/e_ is differently defined for internal and external damping, as follows:

| $\text{Ω}_{\text{i}}\text{ }\text{= }\frac{\text{ρ}\text{A}\text{ω}^{2}}{\text{EI}\text{ }\text{+}\text{ }\text{i}\text{ω}{\text{ }\text{C}}_{\text{i}}}\text{,}$ | (A.11) |
| --- | --- |
| $\text{Ω}_{\text{e}}\text{ }\text{=}\text{ } \frac{\text{ρ}\text{A}\text{ω}^{2}\text{ }-\text{ i}\text{ω}\text{ }\text{C}_{\text{e}}}{\text{EI}} .$ | (A.12) |

The solution to Eqs. (A.7) and (A.8) reads,

| $\text{w}\left( \text{z} \right)\text{ =}\text{ A}_{1}\cos\left( \text{Ω}_{\text{i/e}}\text{z} \right)\text{ + }\text{A}_{2}\sin\left( \text{Ω}_{\text{i/e}}\text{z} \right)\text{ +}\text{ A}_{3}\cosh\left( \text{Ω}_{\text{i/e}}\text{z} \right)\text{ + }\text{A}_{4}\sinh\left( \text{Ω}_{\text{i/e}}\text{z} \right)$. | (A.13) |
| --- | --- |

Hence, the Floquet-Bloch conditions in terms of transversal displacement and rotation are applied to each side - *l/r* - of the *j^th^* support as,

| $\text{w}_{\text{j+1}}\text{ }\text{=}\text{ }\text{w}_{\text{j}}\text{e}^{\text{μ}}$, $\text{w}_{\text{j-1}}\text{ }\text{=}{\text{ }\text{w}}_{\text{j}}\text{e}^{\text{-μ}}$, |  |
| --- | --- |
| ${\text{φ}^{\text{l}}}_{\text{j+1}}\text{ =}{\text{φ}^{\text{l}}}_{\text{j}}\text{e}^{\text{μ}}$, ${\text{φ}^{\text{l}}}_{\text{j-1}}\text{ = }{\text{φ}^{\text{l}}}_{\text{j}}\text{e}^{\text{-}\text{μ}}$, | (A.14) |
| ${\text{φ}^{\text{r}}}_{\text{j+1}}\text{ =}{\text{ φ}^{\text{r}}}_{\text{j}}\text{e}^{\text{μ}}$, ${\text{φ}^{\text{r}}}_{\text{j-1}}\text{ =}{\text{ φ}^{\text{r}}}_{\text{j}}\text{e}^{\text{-μ}}$, |  |

where *l* and *r* mean left and right side, respectively. To compute the constants in Eq. (A.13), we apply the kinematic compatibility and equilibrium conditions at the *j^th^* support. Such conditions lead to a system of linear homogeneous equations^15^ in terms of transversal displacement *w_j_* and rotation at the right side *φ^r^_j_* of the support. After obtaining the *2x2* matrix representing the system and setting its determinant to be non-zero, the dispersion relation of a periodic piping system supported by flexible springs is obtained as,

| $\text{ψ}_{\text{i/e}}{\text{ }\text{cosh}}^{2}\text{(}\text{μ}_{\text{i/e}}\text{) }\text{+}\text{ }\text{χ}_{\text{i/e}}\text{ }\text{cosh}\text{(}\text{μ}_{\text{i/e}}\text{) }\text{+}\text{ }\text{η}_{\text{i/e}}\text{ }\text{=}\text{ }0$, | (A.15) |
| --- | --- |

in which *ψ_i/e_* is defined as follows:

| $\text{ψ}_{\text{i/e}}\text{ }\text{=}\text{ }\left[ {\text{\{cosh}\left( \text{Ω}_{\text{i/e}}\text{l} \right)\text{-cos(}\text{Ω}_{\text{i/e}}\text{l}\text{)\}]}}^{2}\text{-}\text{ }\left\{ \text{sinh}^{2} \left( \text{Ω}_{\text{i/e}}\text{l} \right)\text{-}\text{sin}^{2} \left( \text{Ω}_{\text{i/e}}\text{l} \right) \right\} \right]$, | (A.16) |
| --- | --- |

and where *χ_i/e_* and *η_i/e_* are:

| , | (A.17) |
| --- | --- |
| . | (A.18) |

### *Appendix B - Some considerations about the transient analyses and the chaotic behaviour*

We report and comment herein, the results of a few nonlinear transient analyses performed on the single span (SS) model depicted in Fig. 3, with *gap* = 64 mm and *COR* = 0.49, that is, the Optimal solution #1.

Fig. B1 depicts the displacement responses, and the relevant Fourier transforms for the 7^th^ ground motion listed in Tab. 2.

| *(a) (c)*  *(b)* |
| --- |

Fig. B1 – Displacement x(t) of: (a) Node g-5 and f-3; and (b) Node g-5 and f-1; (c) FRF of the three nodes. The black solid horizontal lines indicate the gap, whereas the vertical dashed line indicates the first natural frequency ω_pr,1_ of the system, see Fig. 8. The input is the 7^th^ ground motion of Tab. 2.

Only the node *g-5* experiences the nonlinear boundary conditions, i.e., the amplitude constraints highlighted by solid black horizontal lines of Fig. B1 (a) and (b). As expected, the impacts activate higher modes, see Fig. B1 (c); however, these occurrences also affect the neighboring nodes. The amplitude of the vibrations of Node f-3, in fact, exceeds the *gap*, and the relevant *FRF* carries energy over the higher frequencies.

Fig. B2 reports the results of further nonlinear transient analyses obtained with single harmonic excitations.

| *(a)* 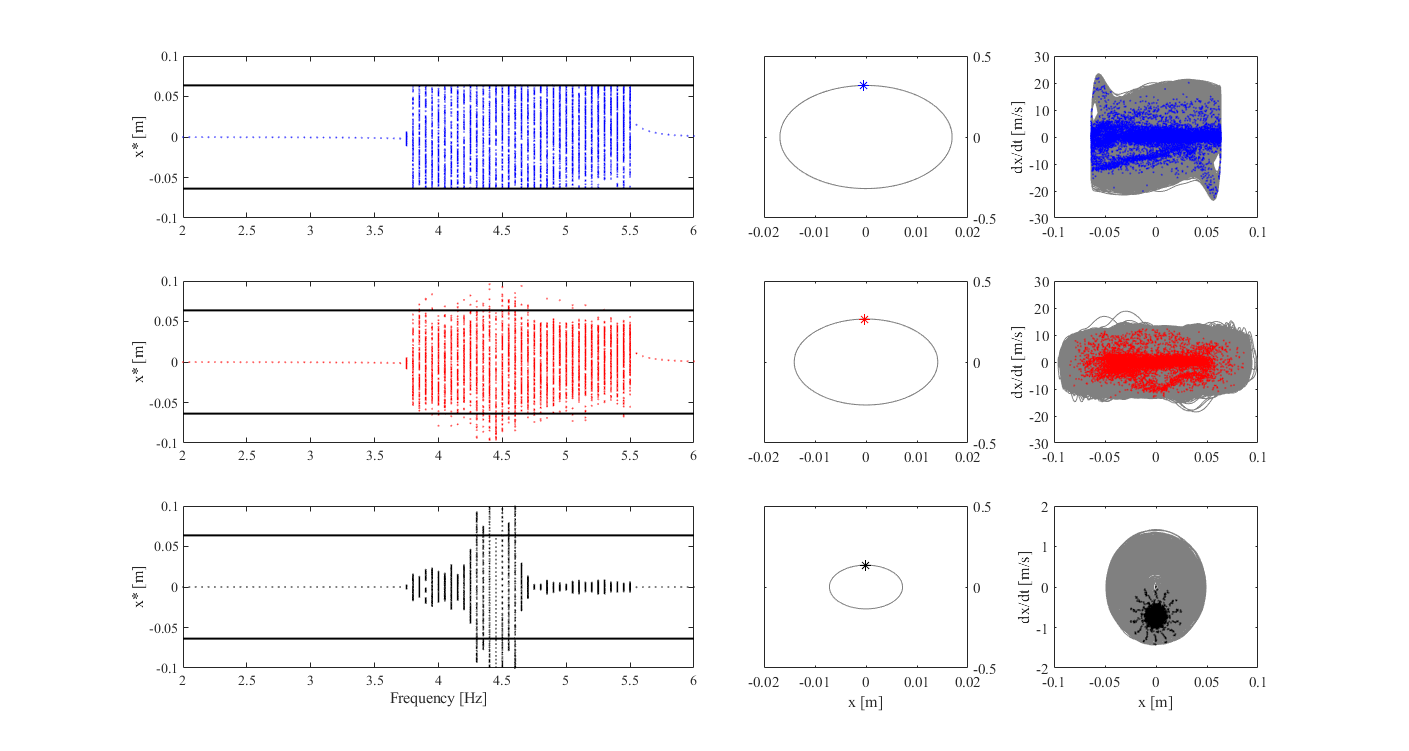 *(b)*  *(c)* |
| --- |

Fig. B2 – From the left to the right-side, respectively: bifurcation diagrams, closed orbits for f_p_ = 3Hz, and phase portraits for f_p_ = 4.8Hz of: (a) Node g-5; (b) Node f-3; and (c) Node f-1. The stars in the closed orbits as well as the dots in the phase portraits report the Poincaré sections. In the phase portraits, the relevant largest Lyapunov exponents λ are reported.

The in-depth characterization of the dynamics of the system requires single harmonic excitations with multiple amplitudes since the dispersion features of a nonlinear system are amplitude and frequency dependent. However, to test the existence of chaotic motion, the periodic forcing of the system has been considered for a limited range of frequencies. The input acceleration reads , where A_p_ = 3 m/s^2^ and *f_p_* ranges from 2 to 6 Hz. The bifurcation diagrams in Fig. B2 report the stable values x* that are approached asymptotically. When the impact does not occur, the system is linear and periodic, for example for *f_p_ = 3* Hz; see, in this respect, Eqs. (11), (12) and (13). Whenever the impact occurs, as for *f_p_ = 4.8* Hz, non-periodic solutions are found, and the attractors are chaotic. The nodes f-1 and f-3, that are not constrained, also experience a chaotic response. We calculated the largest Lyapunov exponent *λ* of the trajectories of Fig. B2 (a), (b) and (c), as follows,

|  | (B.1) |
| --- | --- |

where *K_min_* and *K_max_* represent the range of expansion used to estimate the local expansion rate and *dt* is the sampling time. *Λ_i,K_* defines the logarithmic convergence or divergence of each point in the expansion range, and reads,

|  | (B.2) |
| --- | --- |

where *Y* is the delayed reconstruction of the original signal, with an embedding dimension equal to *4* and a time lag equal to *8*. The *i* indicates that the algorithm seeks for the nearest neighbor *i** that minimizes the denominator of Eq. (B.2) such that *|i-i*|* > *1/f_m_*, where *f_m_* is the mean frequency of the signal. For detailed information about the algorithm, see Rosenstein et al.^41^. As a result, the Lyapunov exponents *λ* for the nodes *g-5*, *f-3* and *f-1* have been found to be equal to *1.1168*, *1.0988* and *0.5874*, respectively. These values clearly indicate exponential divergence of two nearby trajectories for slightly different initial conditions.
